# Genome wide association study of agronomic and seed traits in a world collection of proso millet (*Panicum miliaceum* L.)

**DOI:** 10.1186/s12870-021-03111-5

**Published:** 2021-07-09

**Authors:** Sameh Boukail, Mercy Macharia, Mara Miculan, Alberto Masoni, Alessandro Calamai, Enrico Palchetti, Matteo Dell’Acqua

**Affiliations:** 1 Institute of Life Sciences, Scuola Superiore Sant’Anna, Pisa, Italy; 2 School of Agriculture, University of Florence, Florence, Italy

**Keywords:** Proso millet, Seed morphology, Population genetics, Agrobiodiversity, GWAS, NUC

## Abstract

**Background:**

The climate crisis threatens sustainability of crop production worldwide. Crop diversification may enhance food security while reducing the negative impacts of climate change. Proso millet (*Panicum milaceum* L.) is a minor cereal crop which holds potential for diversification and adaptation to different environmental conditions. In this study, we assembled a world collection of proso millet consisting of 88 varieties and landraces to investigate its genomic and phenotypic diversity for seed traits, and to identify marker-trait associations (MTA).

**Results:**

Sequencing of restriction-site associated DNA fragments yielded 494 million reads and 2,412 high quality single nucleotide polymorphisms (SNPs). SNPs were used to study the diversity in the collection and perform a genome wide association study (GWAS). A genotypic diversity analysis separated accessions originating in Western Europe, Eastern Asia and Americas from accessions sampled in Southern Asia, Western Asia, and Africa. A Bayesian structure analysis reported four cryptic genetic groups, showing that landraces accessions had a significant level of admixture and that most of the improved proso millet materials clustered separately from landraces. The collection was highly diverse for seed traits, with color varying from white to dark brown and width spanning from 1.8 to 2.6 mm. A GWAS study for seed morphology traits identified 10 MTAs. In addition, we identified three MTAs for agronomic traits that were previously measured on the collection.

**Conclusion:**

Using genomics and automated seed phenotyping, we elucidated phylogenetic relationships and seed diversity in a global millet collection. Overall, we identified 13 MTAs for key agronomic and seed traits indicating the presence of alleles with potential for application in proso breeding programs.

**Supplementary Information:**

The online version contains supplementary material available at 10.1186/s12870-021-03111-5.

## Introduction

Millets are amongst the earliest economically important domesticated crops [1, 2] and still an important staple in the semiarid tropics, especially for smallholder farmers with limited access to inputs necessary to grow major food crops [3]. The term “millets” is a broad definition based on their produce and use; however, they are a heterogenous group of species with different origins and taxonomy. Millets have received scant breeding attention due to limited yield potential in conventional agriculture [4]. Nevertheless, millets are amongst the most promising neglected and underutilized crops (NUC) that may improve food security and nutritional quality if properly valorized [5, 6]. So far, research efforts have mainly focused on pearl millet (*Pennisetum glaucum*) and foxtail millet (*Setaria italica*), both of which have established germplasm resources held at the International Crops Research Institute for the Semi-Arid Tropics (ICRISAT) and complete genome sequences [7, 8]. Other millet species have limited resources available and until recently have been overlooked by modern research methods.

Proso millet (*Panicum miliaceum* L.) is a diploid (2n = 36) annual herbaceous plant grown in Eurasia, Oceania, North America, and more rarely in Africa. It has good adaptability to different environmental conditions and requires low rainfall, it is mainly cultivated in arid climates [9], and has a short phenological cycle of about 12 weeks making it a good resource for multiple rotations [10]. In many agroecologies, proso millet is used primarily as livestock feed but has potential as a source of ethanol and as a food grain [11]. Indeed, proso millet flour is rich in proteins, vitamins, minerals, and micronutrients, including iron, zinc, copper, and manganese [12]. Its grains are richer in essential amino acids than those of wheat [13]. However, despites its health benefits and valuable nutritional composition, there is still a large gap in the knowledge needed to integrate proso in the food industry [14].

Proso millet shows high variation in its morphological features [15, 16]. Early studies categorized proso millet germplasm into five races based on morphology of the panicle: *miliaceum*, *patentissimum*, *contractum*, *compactum*, and *ovatum* [17]. More recent studies assessed proso millet diversity based on morpho-agronomic traits, showing high potential for breeding [9] and high resilience towards temperature and drought stress [18]. Studies on the molecular diversity of proso millet collections are limited and seldom used next generation sequencing (NGS) technologies: genetic diversity studies in proso millet mostly relied on RAPD [19], AFLP [20] and SSR markers [21]. Although some of these markers can support marker assisted breeding [22], NGS-based markers can now be produced with limited costs and effort to markedly increase the definition of the molecular characterization of any germplasm collection [23]. NGS paved the way to genotyping by sequencing approaches aimed at the de novo identification of single nucleotide polymorphisms (SNPs), accelerating the characterization of NUCs. More recently, NGS technologies have been applied for the characterization of proso millet allelic pools, including the study of genome wide diversity [24], characterization of gene expression [25] and even forward genetic approaches to identify quantitative trait loci (QTL) and marker-trait associations (MTAs) [26]. The recently published proso sequence and chromosome assembly [27, 28] projected this species into modern genomics, disclosing a great potential for gene mining [29] and even large-scale genotyping applications including genome wide association studies (GWAS). Large-scale GWAS research relying on NGS data have the potential to accelerate NUC breeding by bringing untapped collections of allelic variation into mainstream research [30].

Developments in genotyping technologies are complemented by phenotyping methods targeted at producing comprehensive and precise characterization data of germplasm collections. These methods include automated phenotyping, that may be employed to measure with high precision complex traits of agronomic relevance including root traits [31], fruit traits [32], and seed traits [33]. Any of these traits can then be combined with SNP data to identify MTAs that underpin genomic loci of interest [34] and thus project their relevance into breeding decisions. Seed traits, for example, are related to yield performance, and can be used as a proxy to breed for more desirable varieties [35, 36]. These approaches may be applied to untapped collections of NUC allelic diversity and accelerate the development of new varieties [37, 38]. Indeed, GWAS has been previously applied to other millets, enhancing the understanding of genotype variation and its association with phenotypes [7, 39, 40].

In this study, we characterized the genetic diversity and seed trait diversity in a world collection of *P. miliaceum* that is highly diversified for its agronomic traits [18]. We used this information to describe the diversity in the collection and to conduct a GWAS, identifying 13 MTAs related to seed and agronomic traits. Our results support the potential of NGS technologies and automated phenotyping to support breeding of NUCs.

## Results

### Selection of the core collection and phenotypic diversity

A core collection of 88 accessions was selected from a larger proso millet collection to represent different geographic origins and good field performance. Accessions in the core collection came from the following continents: 23 accessions from Eastern Europe, 16 from Western Asia, 14 from Eastern Asia, 12 from Americas, 6 from Southern Asia, 8 from Western Europe, 5 from Africa, 4 from Oceania. Among the accessions, 45 were landraces, 16 varieties, 2 wild forms, and 1 was identified as breeding material. Full information about genetic materials is provided in Additional file 1: Table S1. The core collection was previously characterized for its agronomic performance [18].

**Fig. 1:**
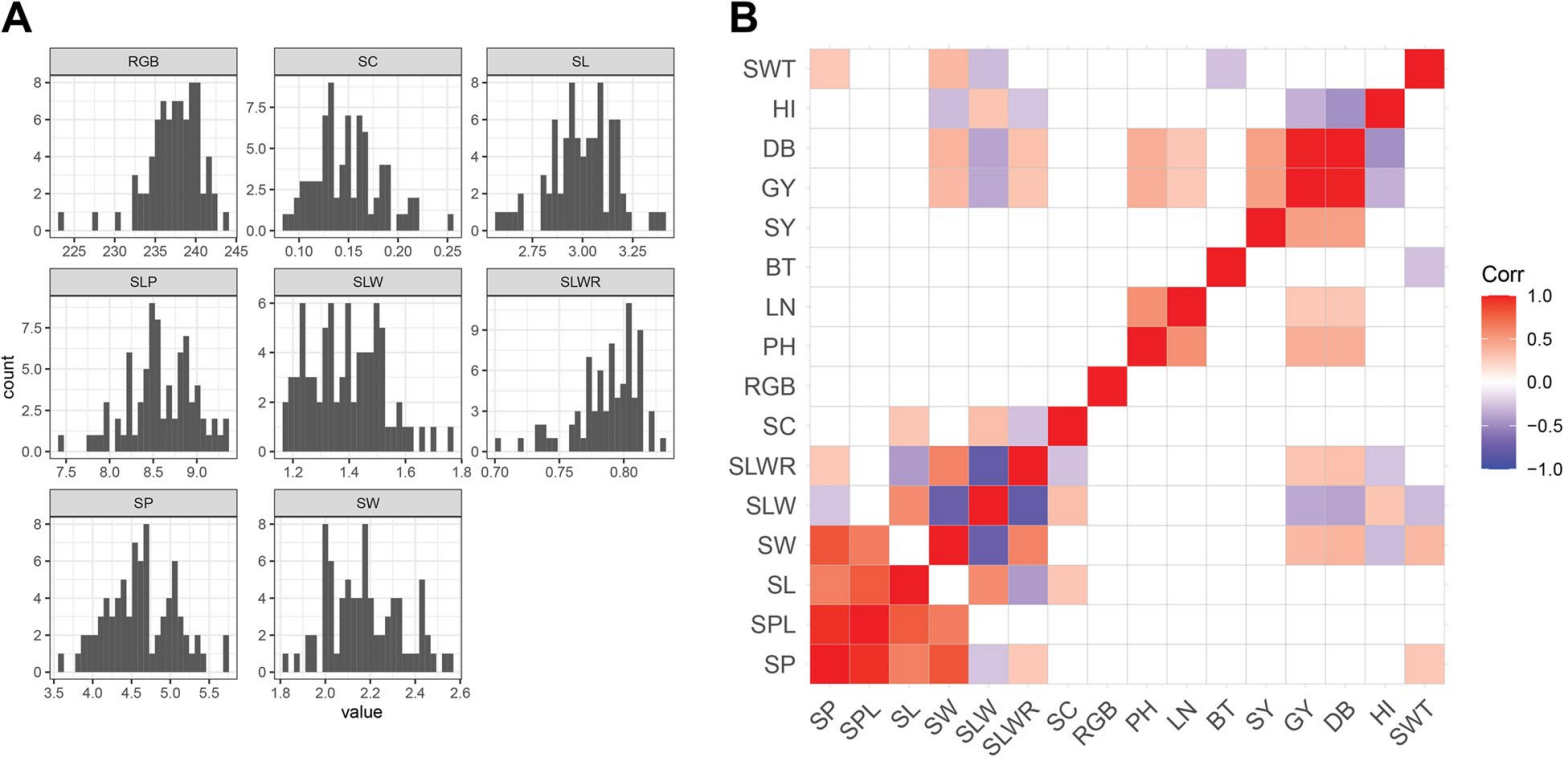
Analysis of seed and morpho-agronomic traits. **A** Histograms for the seed traits. **B** Correlation between seed traits and agronomic traits. The direction and intensity of correlations is shown by the tile colour according to legend. Blank tiles mean no significant correlation. SP, seed perimeter; SPL, seed perimeter to length; SL, seed length; SW, seed width; SLW, seed length to width; SLWR, seed length to width ratio; SC, seed circularity; RGB, seed color; PH, plant height; LN, leaf number; BT, basal tiller number; SY, seed yield; GY, grain yield; DB, dry biomass; HI, harvest index; SWT, seed weight

We selected healthy seeds from the same harvest for each accession and we performed automated phenotyping on seed measures including seed length (SL, in mm), seed width (SW, in mm), seed perimeter (SP, in mm), seed perimeter to length (SPL, in mm), seed length to width (SLW, in mm), seed length to width ratio (SLWR), seed circularity (SC), and seed color (RGB). We found a broad variability for all the seed traits analysed (Table 1). Distributions of phenotype frequencies were mostly normal  (Fig. 1A), even though SLWR, RGB and SC showed excess kurtosis because of uneven distribution of seed shapes in the collection (Additional file 2: Figure S1). A correlation analysis was performed among all measured traits. As expected, most of the seed size traits were highly correlated, but no correlation was observed between seed size and color (Fig. 1B). When seed traits were correlated with agronomic traits previously measured on the collection [18], we identified significant positive correlations between SW, SWT and dry biomass (DB) and grain yield (GY), meaning that accessions with larger seeds had higher yield (Fig. 1B). An analysis of variance (ANOVA) indicated that accessions from the Americas had a different shape with largest SW and a significantly lower SLW and SLWR; all these accessions were improved materials (Additional file 2: Figure S2). Seed accessions from Western Asia were significantly smaller as reported by SW, with most of them being landrace materials. Based on visible differences in seed colour, the collection could be divided into five groups: 52 (59.09%) yellow, 15 (17.04%) white/grey, 7 (7.95%) green, 7 (7.95%) orange/red, and 7 (7.95%) black. RGB values in scanned pictures confirmed a higher number of lighter seeded accessions, with Western and Southern Asia having significantly different RGB values from the rest (Additional file 2: Figure S2).

**Table 1:** Summary statistics for seed phenotypic traits. For each trait, the table reports the average (Mean), standard deviation (Sd), minumum (Min) and maximum (Max) values measured in the collection

Summary statistics	SP	SPL	SL	SW	SLW	SLWR	SC	RGB
Mean	4.6	8.57	2.99	2.17	1.39	0.79	0.15	237.2
Sd	0.43	0.37	0.16	0.16	0.13	0.02	0.03	3.208
Min	3.58	7.47	2.61	1.82	1.17	0.7	0.09	223.67
Max	5.71	9.36	3.41	2.55	1.75	0.83	0.25	244.04

*SP *Seed perimeter, *SPL *Seed perimeter to length, *SL* Seed length, *SW* Seed width, *SLW* Seed length to width, *SLWR* Seed length to width ratio, *SC* Seed circularity, *RGB* Seed color

**Fig. 2:**
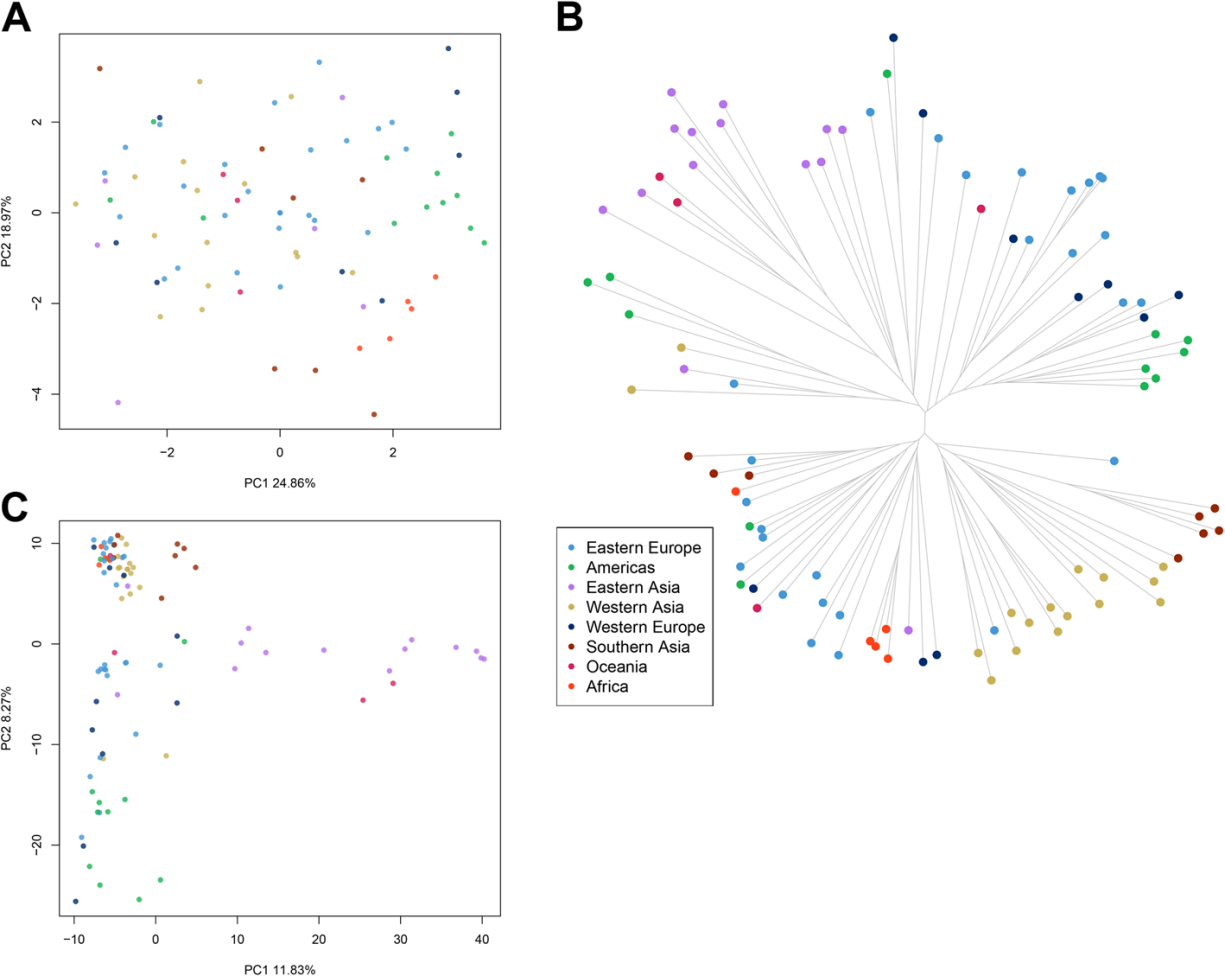
Phenotypic and molecular diversity of proso millet accessions. **A** Principal component analysis of phenotypic diversity of seed traits and agronomic traits. **B** Phylogenetic tree derived from SNPs data. **C** Principal component analysis derived from SNPs data. Different colors indicate region of origin as shown in the legend

A principal component analysis (PCA) was performed on seed and agronomic traits to verify the existence of any structure within the core collection. PC1 and PC2 explained 25% and 19% of the phenotypic variance, respectively, and there was not any clear grouping related to geographic provenance (Fig. 2A). Most landraces and improved material had little to no overlapping (Additional file 2: Figures S3A and S4). The variables most associated with PC1 were SLP, SP, SW (0.24, 0.35 and 0.44 respectively) and negatively SLW, while PC2 was mainly contributed by SLP and SL (0.15 and 0.46 respectively), and negatively by PH (Additional file 2: Figure S5).

### Sequencing and genotypic diversity

DNA was extracted from seedlings of the core collection and sequenced with Illumina technology. Sequencing produced a total of 494.2 M raw reads, 0.67% of which were dropped during adapter removal and quality trimming steps. Mean of retained reads per sample was around 5.14 M. Reads were aligned to the proso millet reference genome obtaining a high proportion of mapped reads (Additional file 1: Table S2). SNP calling resulted in 4,907 good quality markers distributed along all chromosomes, that were further reduced to 2,412 high quality SNPs after stringent filtering to gain in reliability of the allele calls. The phylogenetic tree derived from the set of high-quality SNPs could be grouped in two main clusters (hereafter named Cluster I and Cluster II) (Fig. 2B). Cluster I grouped together samples from Eastern Asia, Americas, and Oceania. Cluster II grouped together the majority of accessions from Western Asia, Southern Asia, and Africa. Accessions from Eastern and Western Europe were fairly distributed between the two clusters. When we compared information on region of origin with type of genetic material, most of the improved accessions grouped into Cluster I with exception of three accessions from eastern Europe (Additional file 2: Figure S3B). Accessions designated as unknown almost always clustered tightly with landraces in both clusters; most were grouped in Cluster II. The two wild accessions clustered in Cluster II (Additional file 2: Figure S3B).

**Fig. 3:**
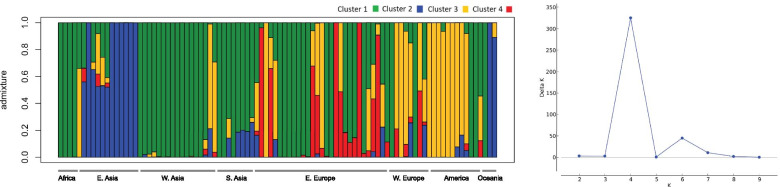
Bayesian structure analysis of the core collection of proso millet. Bar plot representing accession ancestries according to the most probable Structure model (K = 4). Each accession is represented by a vertical bar with colors proportional to their ancestry to one of K genetic cluster according to legend. The panel to the right reports the likelihood of each K interpretation as revealed by the ΔK output from structure Harvester

To better visualize the genetic relationship among individuals, we performed a PCA on molecular data (Fig. 2C; Additional file 2: Figure S3C). The first and the second PCs accounted for 11.83% and 8.27% of the variance, respectively, reporting geographical structure in the dataset particularly among accessions from Eastern Europe, Americas and Eastern Asia which showed little to no overlap in the three PCs (Additional file 2: Figure S6). Samples from Southern Asia and Western Asia consistently grouped together (Fig. 2C). A Bayesian structure analysis revealed that the most probable number of K genetic clusters present in the collection was four (Fig. 3). Overall, results showed that accessions had various degrees of admixture. Accessions from Western Asia, Americas, Southern Asia, and Western Europe had unique background admixture for each region. Oceania samples had a background similar to Western Asia, and African accessions showed no admixture, with a similar genetic background observed in some accessions from Eastern Europe and Western Asia (Fig. 3).

### Genome wide associations of seed and agronomic traits

**Fig. 4:**
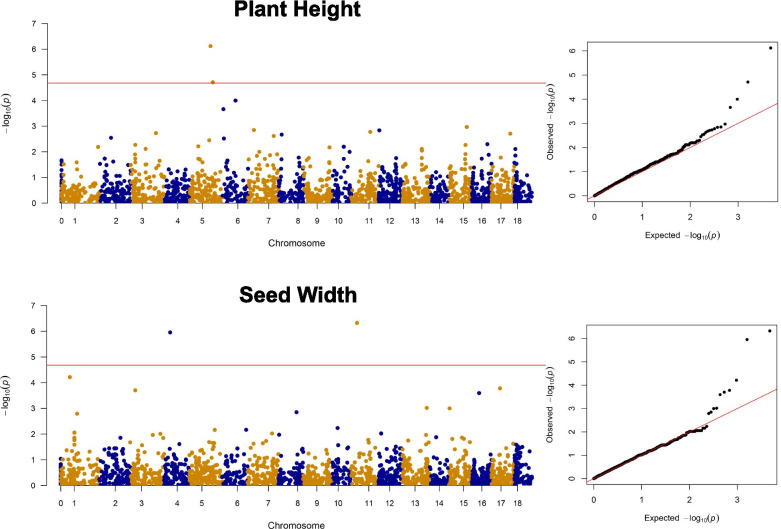
GWAS outcome for plant height and seed width. In the panels to the left, Manhattan plots report individual SNPs across all chromosomes (x-axis) and -log10 *P* value of each SNP association (y-axis). The horizontal lines represent a stringent Bonferroni threshold for a nominal p-value of 0.05. Note that MTAs are called with and FDR-based threshold. Trait names are given on top. The panels to the right report Quantile–Quantile (Q–Q) plots showing distribution of estimated versus observed -log10 (*P*) values obtained by the GWAS model for the traits reported

A GWAS was performed combining phenotypic data with SNP data (Additional file 1: Table S3). Overall, 13 MTAs surpassed the multiple-testing corrected significance threshold, 10 for seed-related traits and three for agronomic traits. For SP, two MTAs were identified at 43.4 Mb and 22.4 Mb, on chromosome (Chr) 6 and Chr 8. Two MTAs were found for SW at 10.5 Mb on Chr 4 and 11.1 Mb on Chr 11. Two MTAs for SL were mapped at 31.6 Mb on Chr 8 and 26.2 Mb on Chr 13. Two additional MTAs were mapped for SLWR at 44.08 Mb on Chr 5 and 43.5 Mb on Chr 6. RGB was associated with two MTAs at 54.1 Mb on Chr 1 and 37.2 Mb on Chr 8. For PH, we identified two MTAs both on chromosome 5 at 35.9 Mb and 40.1 Mb. One MTA was identified for LN at 1.03 Mb on Chr 16 (Table 2; Fig. 4; Additional file 2: Figure S7). MTAs for LN, RGB and SP had the highest significance (Table 2). MTAs associated to PH had the highest effect, providing 9.6 cm and 7.89 cm of additional height, respectively.

**Table 2:** GWAS analysis results. For each trait, the table reports MTAs with their corresponding Chromosome and SNP position, the minor allele frequency (MAF) at the marker, and the effect estimated by the model (in trait units). MTAs all correspond to an FDR adjusted *p* value for multiple testing lower than 5%

**Trait**	**Chromosome**	**SNP Position**	**MAF**	**Effect**
**PH**	5	35,949,582	0.09	-9.65
	5	40,102,196	0.19	7.89
**LN**	16	1,030,350	0.08	1.10
**SP**	8	22,443,560	0.18	0.21
	6	43,445,565	0.22	-0.16
**SL**	8	31,615,399	0.18	0.10
	13	26,298,111	0.08	-0.13
**SW**	11	11,145,652	0.10	0.15
	4	10,506,174	0.04	-0.11
**SLWR**	6	43,587,035	0.31	0.01
	5	44,088,380	0.08	-0.01
**RGB**	1	54,112,377	0.12	3.14
	8	37,217,368	0.03	-4.89

## Discussion

In this study, we assembled a collection of 88 proso millet accessions from eight world regions to combine their agronomic and seed traits diversity with their genetic diversity for GWAS. We found high variation and strong association signals with some of the traits, supporting the use of genomics and phenotypic screening to rapidly detect MTAs with the potential to accelerate NUCs breeding.

For our phenotyping characterization, we focused on quantitative traits of seed size, shape, and colour. These traits have high breeding relevance, as they reflect genetic, physiologic, and ecologic variation that exceed seed morphology [41, 42]. Indeed, seed morphology can influence germination physiology, nutrient quality, and yield [43, 44]. Seed traits may be related to adult plant traits that play an important role in the crop life cycle and environment adaptability and are a component of yield potential [45–47]. Yield components are a valuable target for breeding effort, especially for NUCs with limited story of genetic improvement. Targeting these traits provides several advantages, including the fact that they typically have a simpler genetic determination than yield, are easier to measure, and are less influenced by the environment, resulting in higher heritability [48]. These features make these traits valuable targets for marker assisted selection. Finally, seed traits are easy to phenotype due to availability of automated methods, making the characterization of large ex situ collections possible without the need for field experiments [49]. Phenotyping in open fields is influenced by environmental factors affecting the performance of genotypes, making GWAS more challenging. For instance, the size of inflorescence and number of spikelets in proso millet is highly influenced by the photoperiod, with short days inducing a reduction of both traits [50]. Although quantifying environmental effects is a valuable tool for breeding, imaging-based phenotyping of seeds may provide key information to prioritize genetic materials. Indeed, genomic regions associated with grain size have been previously identified in other cereals including rice [51] maize [52] wheat [53], and barley [54].

The genotyping of the core collection using a NGS allowed us to develop a high-quality set of genome wide SNPs, shedding light on the phylogenetic relationships in global proso millet. Few landraces clustered with improved material suggesting minimal departure from breeding to traditional varieties from which they derive. The grouping of accessions depending on their genotypic diversity (Fig. 2C; Additional file 2: Figure S3C) did not correspond to any clear grouping of accessions based on their phenotypic diversity (Fig. 2A; Additional file 2: Figure S3A). Still, the phenotypic and molecular diversity reported in the proso millet collection suggest a high potential for use in future breeding programs. Our results show that accessions sourced in Eastern Asia (China, Korea, Japan, and Taiwan) are different from accessions in Western Asia (Afghanistan, Iran, Iraq, Kyrgyzstan and Kazakhstan) and Southern Asia (India and Nepal) (Fig. 2B). This finding corresponds to previous studies reporting two main groups in proso millet from Asia: one group including Eastern Asiatic countries, including China, and another group including Western Asiatic countries [55]. Although breeding materials are often exchanged between countries, landraces are unlikely to be transferred over great geographic distances. Their historic association with a specific locality may date back hundreds or even thousands of years [56, 57], allowing to reconstruct differentiation that resulted from past processes. Two hypotheses can be made to define the origin and spread of proso millet; either (1) a single center of origin in China followed by its spread westward, with proso reaching central Asia prior to its arrival in eastern Europe, or (2) multiple domestication centers [55]. It is known that migrations across the inner Asian mountain corridor after third millennium BC could have contributed to the spread of several cereals including proso millet [58]. Our genomics data support this hypothesis, grouping Western Asian accessions with European accession in Cluster II, although more studies considering broader germplasm collections are needed to fully unravel millet evolutionary history.

The GWAS provided strong association signals supported by good model fits as reported by QQ-plots (Additional file 2: Supplementary S8). Genetic maps previously developed for proso millet are fractured and not assembled in chromosomes [26] and limit the possibility to evaluate the co-mapping of our MTAs with previous results. Yet, the strength and magnitude of the associations that we report are suggestive of MTAs with relevance in proso millet genetic improvement. Most accessions (~ 76%) in our collection had lighter shaded seed. GWAS results revealed two MTAs for RGB on Chr 1 at 54.1 Mb and on Chr 8 at 37.8 Mb. Previous studies have shown that differentiation in seed colour for minor cereals may be due to composition of tannin in the husk, darker seeds in proso millet having highest tannin content [59, 60]. Tannin is a phenolic compound with a high antioxidant potential [61, 62], meaning the darker seeds may have superior nutritional aspects. Regardless, consumers typically prefer yellow-coloured grains [63], and this is reflected to the prevalence of this color in our collection. Interestingly, seed coat colour has been associated with seed germinability and viability in proso millet [64, 65], suggesting pleiotropy with other important traits not considered in this study.

Natural selection favours round and small size in wild relatives, but breeding has always focused on increasing seed size [66]. We identified eight MTAs for seed size traits SP, SL, SW and SLWR. We found positive correlations among these seed size traits (Fig. 1B) yet limited overlap in the MTAs observed for these traits. We found MTAs on the same chromosome for SP and SL on Chr 8, 9.17 Mb apart and SP and SLWR on Chr 6, 0.14 Mb apart, suggesting the presence of linked genomic loci controlling these traits. In millets other than proso, genome wide studies have identified QTL for seed size traits including grain yield and seed weight on Chr 4, 5 [67], Chr 1, 2, 3, 6, 7 [68], Chr 2, 3, 6, 8, 9 [69], and Chr 3, 4, 5 [70]. Grain size in terms of seed length, width and perimeter has direct impact on seed weight and consequently grain yield; we found a positive correlation of SP, SW with the weight of seeds (SWT) (Fig. 1B) but no overlapping MTAs. Among the significantly associated SNPs, we identified four MTAs for seed size traits on Chr 6 and Chr 8. In foxtail millet, signals on Chr 6 and 8 were put in relation with seed weight traits including thousand grain weight (TGW) and grain yield (GY) [69, 71].

We found a positive correlation of LN with PH, GY and DB (Fig. 1B) and one MTA on Chr 16 with an estimated effect of 1.1 additional leaves LN. QTL for leaf traits have been extensively described in other crops including *Triticum aestivum* [72], *Elaeis guineensis* [73] and *Hordeum vulgare* [74] as a gateway for improvement of plant architecture. Plant height in our collection is quite diversified and ranges from 30 to 100 cm [75]. Breeding for PH is crucial as this trait is strongly correlated with reproduction, seed mass, yield, and rate of maturity [76]. In the present study, we found two MTAs for PH on Chr 5 located 4.15 Mb apart and explaining a large variation in the trait (Table 2). Plant height has previously been mapped on Chr 5 in foxtail millet (*Setaria italica*) [70, 77, 78], whose genome has an estimated > 85% transferability to other small millets including proso [6]. Leaf number plays a direct role in a plant’s photosynthetic ability and consequently contributes to yield [79, 80].

## Conclusion

Our characterization revealed broad diversity for seed and molecular traits in proso millet germplasm, a valuable resource to accelerate breeding in this species. Although the size of the population used in the present study is small, we identified significant MTAs even after implementing a stringent multiple testing correction. Further studies may focus on larger collections and multiple-years phenotyping experiments to increase the depth of the characterization reported here. The accumulation of layers of information on the genomics tools available on millet will further increase the exploitability of our results. This includes the development of markers such as Kompetitive Allele Specific PCR (KASP) that may be readily used to support marker assisted selection in breeding. KASP developed for proso millet MTAs could then be used to screen for presence of these loci in large millet collections. As more information will be available, molecular studies can focus on the validation of MTAs to understand the molecular mechanisms underpinning seed and agronomic traits. Currently, our results report high potential for breeding in proso millet plant genetic resources, supporting the use of NGS and automated phenotyping to propel breeding efforts on this NUC.

## Materials and methods

### Selection of core collection

Seeds of 300 *P. miliaceum* (L.) accessions were obtained from the United States Department of Agriculture (USDA) germplasm bank. No special permissions were necessary to collect samples. In 2016, the full collection was sown for seed multiplication and screening in open field at San Piero a Grado, Pisa, Italy (43.6797°N, 10.3468°E) using mulching with plants 50 cm apart to avoid cross-pollination. A core collection was derived from the full collection so that the resulting pool of samples would summarize world proso millet diversity. The passport data available for each accession was obtained from USDA. Priority was given to accessions having i) a known geographic origin, ii) at least partial information available in their passport data, iii) a good performance shown during the amplification field experiment of 2016. Based on these criteria, a core collection of 88 accessions was selected to undergo seed phenotyping and genotyping (Additional file 1: Table S1). Formal identification of the samples was performed by the Corresponding Author. No voucher specimens were deposited.

### Seed phenotyping

Fifteen healthy seeds from each accession were selected from the 2016 harvest for seed phenotyping. Seeds were arranged within a cardboard frame and placed on the scanning surface of an Epson Perfection 3170 photo image scanner. Images were scanned at 3200dpi and analyzed using the digital image analysis software SmartGRAIN [81]. Seed detection was manually curated on each accession following the software’s guidelines. A scale was set according to image definition, and seed characterization was run in batch. For each of 15 seeds per accession, the program measured seed perimeter (SP, in mm), seed perimeter to length (SPL, in mm), seed length (SL, in mm), seed width (SW, in mm), seed length to width (SLW, in mm), seed length to width ratio (SLWR) and seed circularity (SC). Seed color (RGB) index was measured using ImageJ software version 1.52A [82]. The software was run in batch to calculate the amount of red, green, blue (RGB) light emitted for each pixel in each of the scanned images. As the phenotyping chamber was constant, any difference in RGB was attributed to differences in seed color. The resulting RGB value was used as a phenotypic measurement.

In a previous experiment, the same proso accessions were planted at the Agricultural Institute of Florence, Italy (40°58′N, 14°14′ E) and agronomic traits were recorded: these included plant height (PH, cm), leaf number (LN), basal tiller number (BT), seed yield (SY, kg ha^−1^), grain yield (GY, kg ha^−1^), dry biomass (DB, kg ha^−1^), harvest index (HI, grain / biomass) and seed weight (SWT, g/100). Full details are given in [18].

### Genotyping

In 2017, seeds from the imaging analysis were germinated in petri dishes, and green leaves were collected and pooled from five seedlings. Genomic DNA was extracted using the GenElute Plant Genomic DNA Miniprep Kit (Sigma Aldrich, Germany) following the manufacturer’s instructions. Genomic DNA integrity was evaluated in 1% agarose gel and quantified using the Qubit fluorometer (Invitrogen, Thermo Fisher Scientific, US). Sequencing was conducted at IGA Technology Services (Udine, Italy). Sequencing libraries were prepared according to the restriction-site associated DNA marker (RAD) protocol [83] using the *HindIII* restriction enzyme. RAD libraries were multiplexed and sequenced on an Illumina HiSeq 2000 machine to produce short reads which were then de-multiplexed and checked for quality with FastQC [84]. Briefly, raw reads were filtered using ERNE-FILTER v.2.1.2 (http://erne.sourceforge.net/) [85]. Filtering criteria followed standard procedures to ensure only high-quality reads were retained. Filtered reads were mapped against the proso millet reference genome assembly version GCA_003046395.2 (NCBI identifier: PRJNA431363) using BWA-mem algorithm v0.7.17 (https://github.com/lh3/bwa/releases/tag/v0.7.17) with default parameters.

SNPs calling was conducted using the HaplotypeCaller [86] from Genome Analyzer Tool Kit package version 4.2.0.0 in GVCF mode (https://github.com/broadinstitute/gatk/releases), following the best practice. Only high quality (QUAL > 30.0) biallelic SNPs were retained. Finally, SNPs with minor allele frequency (MAF) lower than 1% were removed using Tassel 5.0 [87] and R [88] custom scripts. All raw reads are available at the National Center for Biotechnology Information (NCBI) database (https://www.ncbi.nlm.nih.gov) under BioProject ID PRJNA726150.

### Data analysis

Seed phenotyping data was analyzed with R [88] using custom scripts. For each measured seed trait, the three highest and three lowest measures among the 15 seeds analyzed per accessions were removed, and an average was computed on the remaining ones. This was intended to remove possible outliers from sub-optimal seed recognition by the imaging software. R/corrplot [89], was used to study the correlation among seeds and plant traits. A PCA was performed to estimate the relative importance of different traits in capturing variation in the collection, and to establish the relationship among all variables under study. An ANOVA (R/ggplot2) was performed to determine statistical significance of diversity in seed trait phenotypic data in the different regions.

Genetic diversity analyses were performed using R [88] to survey different aspects of the molecular diversity in the proso collection and to identify subpopulations. A neighbor-joining phylogeny was produced with R/adegenet [90] and a PCA was performed on the SNPs dataset to survey the existence and distance of genetic clades in the collection. Structure 2.3.4 [91] was used to assign individuals to cryptic genetic clusters, by detecting the number of clusters that best described the data. Structure was run with the admixture model with 10 000 burn-in iterations and 100 000 MCMC repetitions. Parameters were tested from K = 1 to K = 10, where K is the number of genetic groups, with 10 replications each. After running the program, resulting data were loaded in Structure Harvester [92] to produce ad hoc statistics to identify the most probable K according to Evanno method [93].

Phenotypic and genotypic data were combined in a GWAS analysis, adding data from morpho-agronomic diversity previously reported [18]. The GWAS was run using the fixed and random model Circulating Probability Unification (FarmCPU) model [94] implemented in R/GAPIT [95] using the first PC calculated on genotypic data as covariate. MTAs are defined as SNPs surpassing the significance threshold of a false discovery rate (FDR) < 0.05 according to Storey’s method [96]. Plotting of GWAS results was performed with the R package qqman [97]; Manhattan plots display a stringent Bonferroni threshold corresponding to a nominal *p*-value of 0.05 to aid the identification of most significant SNPs.

## Supplementary Information

**Additional file 1: Supplementary Table 1.** Summary of accessions used in this study. The table reports the sample name, the type of material and geographic information on its origin. Accession ID and name report the international identifiers of the sample as reported in the USDA passport information. **Supplementary Table 2.** Overview of RAD sequencing data produced and alignment to the reference sequence. For each sample, the table reports the total amount of reads passing the quality threshold, the total amount of reads mapped on the genome, and the ratio of the two. **Supplementary Table 3.** GWAS Summary data. For each SNP, the table reports the chromosome and position on the reference genome. For each trait-SNP combination, the table reports the p-value of the association and estimated effect. Trait names are coded as in the main text.

**Additional file 2: Supplementary Figure S1.** Representative photos for proso millet seeds color classes characterized in this study. **Supplementary Figure S2.** Boxplots of seed trait distribution across geographical regions. Differences were analyzed using ANOVA. **Supplementary Figure S3.** Phenotypic and molecular analysis of proso millet accessions. (A) Principal component analysis of phenotypic diversity of seed traits and agronomic traits. (B) Phylogenetic tree derived from SNPs data. (C) Principal component analysis derived from SNPs data. Different symbols indicate type of genetic materials as shown in the legend. **Supplementary Figure S4.** Phenotypic diversity in the collection as reported by PC1, PC2, and PC3. Different colors on and symbols on the panels indicate region of origin and type of genetic materials as in Fig. 2 and Supplementary Figure S3. **Supplementary Figure S5.** PCA scores and vectors loadings for seed and agronomic traits. Percent of variance explained by each axis (PC1 = Dim1, PC2 = Dim2) is indicated in the axis titles. Vector color represents the total contribution of a given variable on the first two dimensions according to legend to the right. **Supplementary Figure S6.** Genotypic diversity in the collection as reported by PC1, PC2, and PCA3. Different colors on and symbols on the panels indicate region of origin and type of genetic materials as in Fig. 2 and Supplementary Figure S3. **Supplementary Figure S7.** Manhattan plots for GWAS on seed trait and agronomic traits. The plot shows individual SNPs across all chromosomes (x-axis) and -log10 P value of each SNP association (y-axis). The different colors indicate the 18 chromosomes of proso millet. The horizontal line shows the multiple testing threshold according to a stringent Bonferroni method. Note that different Manhattan plots are reported to different y-axis scales corresponding to the highest significance for each GWAS. **Supplementary Figure S8.** Quantile–Quantile (Q–Q) plots for FarmCPU model showing distribution of estimated versus observed -log10 (P) values for seed trait and agronomic traits.

## Data Availability

All data generated or analysed during this study are included in this published article as supplementary materials. Raw reads are available at the National Center for Biotechnology Information (NCBI) database (https://www.ncbi.nlm.nih.gov) under BioProject ID PRJNA726150. Field phenotypes were derived from reference [18]. Data access for all databeses used is open. Scripts are available contacting the corresponding author.

## Abbreviations

SL: Seed length
SW: Seed width
SP: Seed perimeter
SPL: Seed perimeter to length
SLW: Seed length to width
SLWR: Seed length to width ratio
SC: Seed circularity
RGB: Seed color
PH: Plant height
LN: Leaf number
BT: Basal tiller
SY: Seed yield
GY: Grain yield
DB: Dry biomass
HI: Harvest index
SWT: Seed weight/100
NUC: Neglected and underutilized crops
Chr: Chromosome
