# Supplementary material for: Genome wide association study of agronomic and seed traits in a world collection of proso millet (Panicum miliaceum L.)
Source: BMC Plant Biol. 2021 Jul 9;21:330. doi: 10.1186/s12870-021-03111-5 (PMC8268170; doi:10.1186/s12870-021-03111-5)
Supplement: Supplementary file 1 — Additional file 1: Supplementary Table 1. Summary of accessions used in this study. The table reports the sample name, the type of material and geographic information on its origin. Accession ID and name report the international identifiers of the sample as reported in the USDA passport information. Supplementary Table 2. Overview of RAD sequencing data produced and alignment to the reference sequence. For each sample, the table reports the total amount of reads passing the quality threshold, the total amount of reads mapped on the genome, and the ratio of the two. Supplementary Table 3. GWAS Summary data. For each SNP, the table reports the chromosome and position on the reference genome. For each trait-SNP combination, the table reports the p-value of the association and estimated effect. Trait names are coded as in the main text. [file 12870_2021_3111_MOESM1_ESM.zip › Additional File 1_ Table_S1_ESM.docx]

**Supplementary Table 1: Summary of accessions used in this study.** The table reports the sample name, the type of material and geographic information on its origin. Accession ID and name report the international identifiers of the sample as reported in the USDA passport information.

| **Sample name** | **Type of material** | **County of origin** | **Continent of origin** | **Accession ID** | **Name** |
| --- | --- | --- | --- | --- | --- |
| SMS_002 | Unknown | Georgia | Eastern Europe | Ames 32316 | GE.2013-28 |
| SMS_003 | Wild | France | Western Europe | PI 649372 | Index Seminum 295 |
| SMS_007 | Unknown | India | Southern Asia | Ames 11641 | I.Pm. 630 |
| SMS_009 | Unknown | India | Southern Asia | Ames 11674 | I.Pm. 669 |
| SMS_010 | Unknown | India | Southern Asia | Ames 11678 | I.Pm. 673 |
| SMS_041 | Landrace | Turkey | Eastern Europe | PI 170602 | Harlan 3365 |
| SMS_044 | Landrace | Turkey | Eastern Europe | PI 171727 | DARI |
| SMS_046 | Landrace | Turkey | Eastern Europe | PI 173749 | KIRMIZIDARI |
| SMS_089 | Unknown | Argentina | Americas | PI 202294 | IPM 1036 |
| SMS_090 | Landrace | Argentina | Americas | PI 202295 | IPM 686 |
| SMS_095 | Landrace | Japan | Western Asia | PI 207663 | MOROCCO |
| SMS_102 | Unknown | Afghanistan | Eastern Asia | PI 212862 | IPM 1053 |
| SMS_106 | Unknown | Afghanistan | Eastern Asia | PI 220393 | ARZEN |
| SMS_108 | Landrace | Afghanistan | Eastern Asia | PI 220536 | ARZAN |
| SMS_112 | Landrace | Afghanistan | Eastern Asia | PI 220812 | GAL |
| SMS_117 | Landrace | Afghanistan | Eastern Asia | PI 223791 | TAREQ; ARZAN |
| SMS_120 | Unknown | Afghanistan | Eastern Asia | PI 223794 | ARZAN |
| SMS_123 | Unknown | Iran | Eastern Asia | PI 227245 | ARZAN |
| SMS_131 | Landrace | Iran | Eastern Asia | PI 251388 | IPM 1091-3 |
| SMS_132 | Landrace | Iran | Eastern Asia | PI 251389 | IPM 1092 |
| SMS_133 | Landrace | Iran | Eastern Asia | PI 251403 | ARZAN |
| SMS_136 | Unknown | Iran | Eastern Asia | PI 251406 | ARZAN |
| SMS_138 | Landrace | Iraq | Eastern Asia | PI 253789 | IPM 1102 |
| SMS_139 | Landrace | Iraq | Eastern Asia | PI 253790 | IPM 1103 |
| SMS_171 | Landrace | United Kingdom | Western Europe | PI 290726 | IPM 1128 |
| SMS_172 | Landrace | China | Western Asia | PI 291363 | USSR |
| SMS_173 | Unknown | China | Western Asia | PI 291364 | USSR |
| SMS_174 | Landrace | Canada | Americas | PI 296376 | CROWN |
| SMS_176 | Landrace | Ukraine | Eastern Europe | PI 346934 | PODOLIAN 24/273 |
| SMS_178 | Landrace | Kyrgyzstan | Eastern Asia | PI 346936 | TOKOQULSK |
| SMS_180 | Landrace | Kazakhstan | Eastern Asia | PI 346938 | URAL 1419 |
| SMS_181 | Landrace | Kazakhstan | Eastern Asia | PI 346939 | URAL |
| SMS_183 | Landrace | Ukraine | Eastern Europe | PI 346941 | KHARKOV 25 |
| SMS_184 | Landrace | Ukraine | Eastern Europe | PI 346942 | VESELOPODOLIAN 38 |
| SMS_185 | Landrace | Ukraine | Eastern Europe | PI 346943 | NA |
| SMS_189 | Unknown | Australia | Oceania | PI 365840 | NA |
| SMS_194 | Unknown | Australia | Oceania | PI 365845 | NA |
| SMS_198 | Landrace | Australia | Oceania | PI 367683 | WHITE FRENCH STRN. 8567-7 |
| SMS_199 | Landrace | Australia | Oceania | PI 367684 | WHITE FRENCH COMMERCIAL |
| SMS_201 | Unknown | China | Western Asia | PI 408805 | UI 4825 |
| SMS_202 | Unknown | Nepal | Southern Asia | PI 427247 | NA |
| SMS_203 | Unknown | Nepal | Southern Asia | PI 427248 | NA |
| SMS_204 | Unknown | Nepal | Southern Asia | PI 427249 | NA |
| SMS_208 | Landrace | Taiwan | Western Asia | PI 433381 | Vishenutu |
| SMS_209 | Unknown | China | Western Asia | PI 436622 | Lung Shu no. 5 |
| SMS_210 | Unknown | China | Western Asia | PI 436623 | Lung Shu no. 7 |
| SMS_211 | Unknown | China | Western Asia | PI 436624 | Lung Shu no. 14 |
| SMS_215 | Unknown | Belgium | Western Europe | PI 442533 | NA |
| SMS_640 | Unknown | Soviet Union | Eastern Europe | PI 476399 | Raoluoga |
| SMS_646 | Improved | Romania | Eastern Europe | PI 516181 | MINERVA |
| SMS_647 | Unknown | Morocco | Africa | PI 517016 | GR 656 |
| SMS_648 | Landrace | Morocco | Africa | PI 517017 | GR 658 |
| SMS_649 | Landrace | Morocco | Africa | PI 517018 | GR 664 |
| SMS_650 | Landrace | Morocco | Africa | PI 517019 | GR 665 |
| SMS_651 | Improved | Hungary | Eastern Europe | PI 531397 | BANKUTI FEHER |
| SMS_652 | Landrace | Bulgaria | Eastern Europe | PI 531399 | BOLGAR 161 |
| SMS_653 | Landrace | Hungary | Eastern Europe | PI 531400 | CSASZARRETI 2 |
| SMS_654 | Landrace | Hungary | Eastern Europe | PI 531401 | CSASZARRETI 6 |
| SMS_655 | Landrace | Czechoslovakia | Eastern Europe | PI 531402 | DOMACE BIELE |
| SMS_656 | Landrace | Hungary | Eastern Europe | PI 531403 | DUNAKILITI "A" |
| SMS_659 | Landrace | Czechoslovakia | Eastern Europe | PI 531406 | HANACKE MANA |
| SMS_660 | Landrace | Germany | Western Europe | PI 531407 | HARKOVSKOE 2 |
| SMS_661 | Landrace | Germany | Western Europe | PI 531408 | HARKOVSKOE 65 |
| SMS_665 | Landrace | Germany | Western Europe | PI 531413 | VESZELOPODOLJANSZKOE 403 |
| SMS_668 | Landrace | Hungary | Eastern Europe | PI 531416 | MALCALTOR "A" |
| SMS_670 | Landrace | Hungary | Eastern Europe | PI 531418 | OROSZ-6 |
| SMS_671 | Landrace | Kenya | Africa | PI 531419 | PROSOS |
| SMS_673 | Landrace | Soviet Union | Eastern Europe | PI 531421 | SARATOVSKOE 953 |
| SMS_674 | Landrace | Soviet Union | Eastern Europe | PI 531422 | SARATOVSKOE 953 |
| SMS_675 | Landrace | Poland | Western Europe | PI 531423 | STRELECKIE BRUNATE |
| SMS_679 | Landrace | Soviet Union | Eastern Europe | PI 531427 | TOJDENSKOE 215 |
| SMS_681 | Improved | Soviet Union | Eastern Europe | PI 531430 | VESZELOPODOLJANSZKOE 403 |
| SMS_682 | Improved | United States | Americas | PI 536011 | SUNUP |
| SMS_683 | Improved | United States | Americas | PI 578073 | EARLYBIRD |
| SMS_684 | Improved | United States | Americas | PI 578074 | HUNTSMAN |
| SMS_685 | Unknown | United States | Americas | PI 583347 | SUNRISE |
| SMS_686 | Breeding Material | United States | Americas | PI 583348 | NE1 |
| SMS_687 | Improved | United States | Americas | PI 633425 | Horizon |
| SMS_688 | Wild | Germany | Western Europe | PI 649371 | Index Seminum #568 |
| SMS_691 | Improved | Korea | Western Asia | PI 649375 | Boeun 4 |
| SMS_692 | Improved | Korea | Western Asia | PI 649376 | Cheongsong 4 |
| SMS_693 | Improved | Korea | Western Asia | PI 649377 | Cheongwon 5 |
| SMS_694 | Improved | Korea | Western Asia | PI 649378 | Euiseong 5 |
| SMS_695 | Improved | Korea | Western Asia | PI 649379 | Eumseong 5 |
| SMS_696 | Improved | Korea | Western Asia | PI 649380 | Mooju 6 |
| SMS_699 | Improved | United States | Americas | PI 649383 | Panhandle |
| SMS_700 | Improved | United States | Americas | PI 649384 | Minco |
| SMS_701 | Improved | United States | Americas | PI 649385 | Minsum |
