# Supplementary figures and images for: Genome wide association study of agronomic and seed traits in a world collection of proso millet (Panicum miliaceum L.)
Source: BMC Plant Biol. 2021 Jul 9;21:330. doi: 10.1186/s12870-021-03111-5 (PMC8268170; doi:10.1186/s12870-021-03111-5)

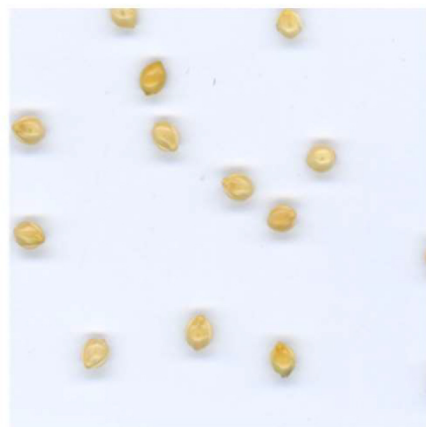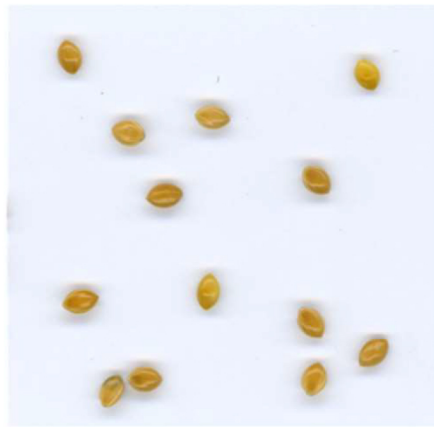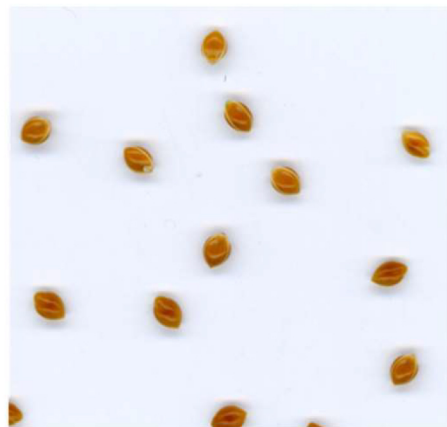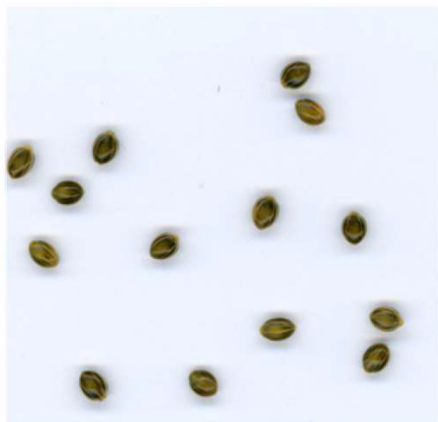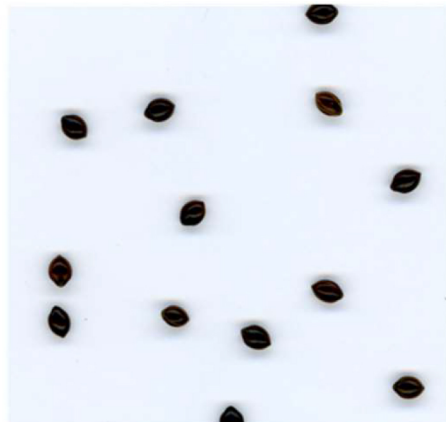

Supplement: Supplementary file 2 — Additional file 2: Supplementary Figure S1. Representative photos for proso millet seeds color classes characterized in this study. Supplementary Figure S2. Boxplots of seed trait distribution across geographical regions. Differences were analyzed using ANOVA. Supplementary Figure S3. Phenotypic and molecular analysis of proso millet accessions. (A) Principal component analysis of phenotypic diversity of seed traits and agronomic traits. (B) Phylogenetic tree derived from SNPs data. (C) Principal component analysis derived from SNPs data. Different symbols indicate type of genetic materials as shown in the legend. Supplementary Figure S4. Phenotypic diversity in the collection as reported by PC1, PC2, and PC3. Different colors on and symbols on the panels indicate region of origin and type of genetic materials as in Fig. 2 and Supplementary Figure S3. Supplementary Figure S5. PCA scores and vectors loadings for seed and agronomic traits. Percent of variance explained by each axis (PC1 = Dim1, PC2 = Dim2) is indicated in the axis titles. Vector color represents the total contribution of a given variable on the first two dimensions according to legend to the right. Supplementary Figure S6. Genotypic diversity in the collection as reported by PC1, PC2, and PCA3. Different colors on and symbols on the panels indicate region of origin and type of genetic materials as in Fig. 2 and Supplementary Figure S3. Supplementary Figure S7. Manhattan plots for GWAS on seed trait and agronomic traits. The plot shows individual SNPs across all chromosomes (x-axis) and -log10 P value of each SNP association (y-axis). The different colors indicate the 18 chromosomes of proso millet. The horizontal line shows the multiple testing threshold according to a stringent Bonferroni method. Note that different Manhattan plots are reported to different y-axis scales corresponding to the highest significance for each GWAS. Supplementary Figure S8. Quantile–Quantile (Q–Q) plots for FarmCPU model s [file 12870_2021_3111_MOESM2_ESM.zip › Fig.S1.pdf]

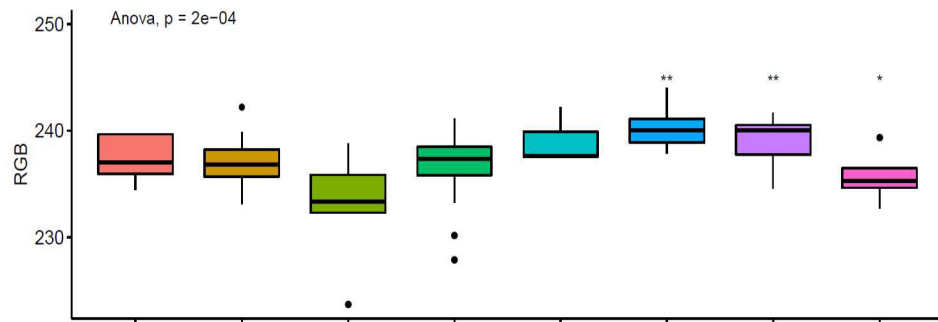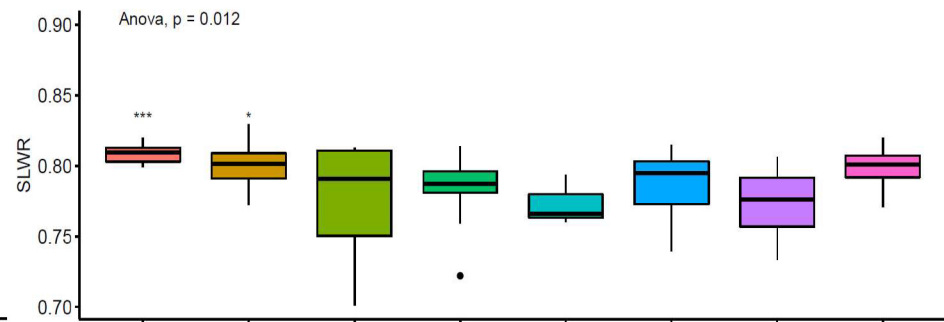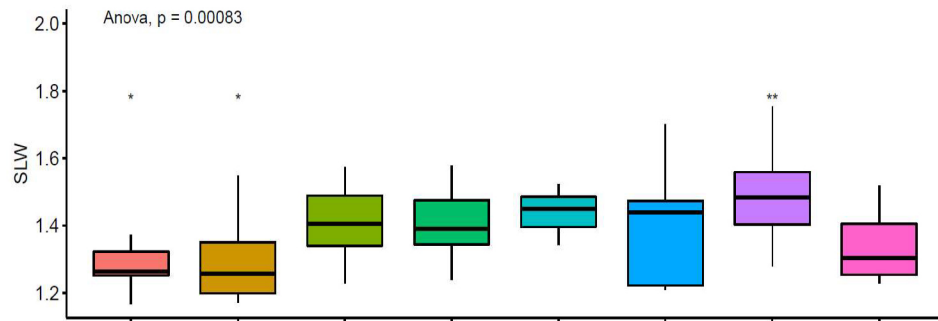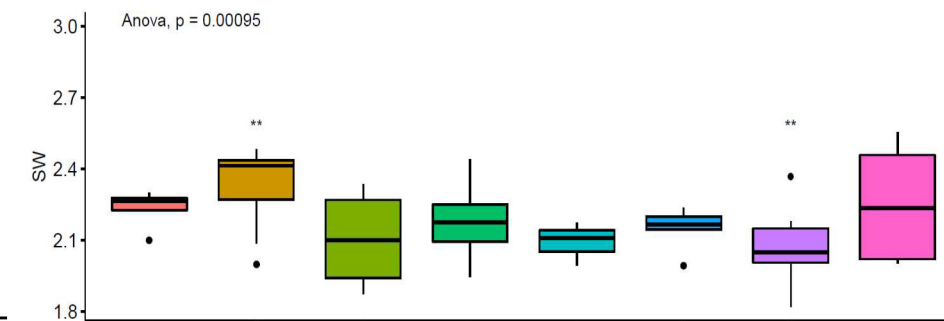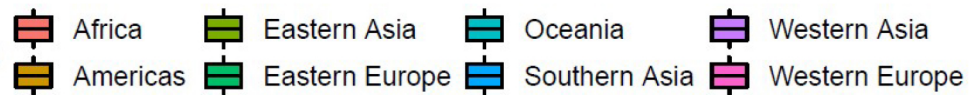

Supplement: Supplementary file 2 — Additional file 2: Supplementary Figure S1. Representative photos for proso millet seeds color classes characterized in this study. Supplementary Figure S2. Boxplots of seed trait distribution across geographical regions. Differences were analyzed using ANOVA. Supplementary Figure S3. Phenotypic and molecular analysis of proso millet accessions. (A) Principal component analysis of phenotypic diversity of seed traits and agronomic traits. (B) Phylogenetic tree derived from SNPs data. (C) Principal component analysis derived from SNPs data. Different symbols indicate type of genetic materials as shown in the legend. Supplementary Figure S4. Phenotypic diversity in the collection as reported by PC1, PC2, and PC3. Different colors on and symbols on the panels indicate region of origin and type of genetic materials as in Fig. 2 and Supplementary Figure S3. Supplementary Figure S5. PCA scores and vectors loadings for seed and agronomic traits. Percent of variance explained by each axis (PC1 = Dim1, PC2 = Dim2) is indicated in the axis titles. Vector color represents the total contribution of a given variable on the first two dimensions according to legend to the right. Supplementary Figure S6. Genotypic diversity in the collection as reported by PC1, PC2, and PCA3. Different colors on and symbols on the panels indicate region of origin and type of genetic materials as in Fig. 2 and Supplementary Figure S3. Supplementary Figure S7. Manhattan plots for GWAS on seed trait and agronomic traits. The plot shows individual SNPs across all chromosomes (x-axis) and -log10 P value of each SNP association (y-axis). The different colors indicate the 18 chromosomes of proso millet. The horizontal line shows the multiple testing threshold according to a stringent Bonferroni method. Note that different Manhattan plots are reported to different y-axis scales corresponding to the highest significance for each GWAS. Supplementary Figure S8. Quantile–Quantile (Q–Q) plots for FarmCPU model s [file 12870_2021_3111_MOESM2_ESM.zip › Fig.S2.pdf]

**A**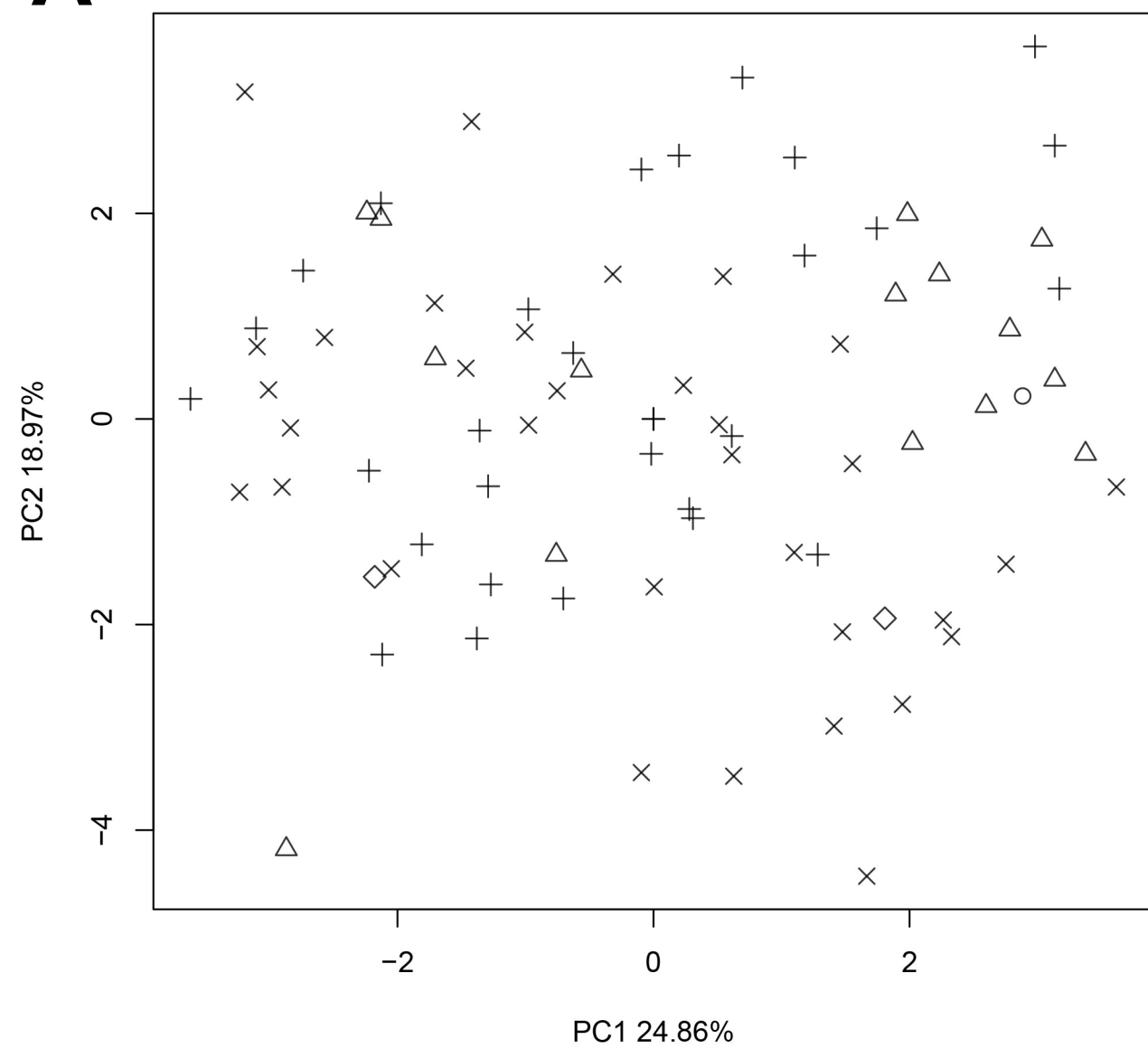**B**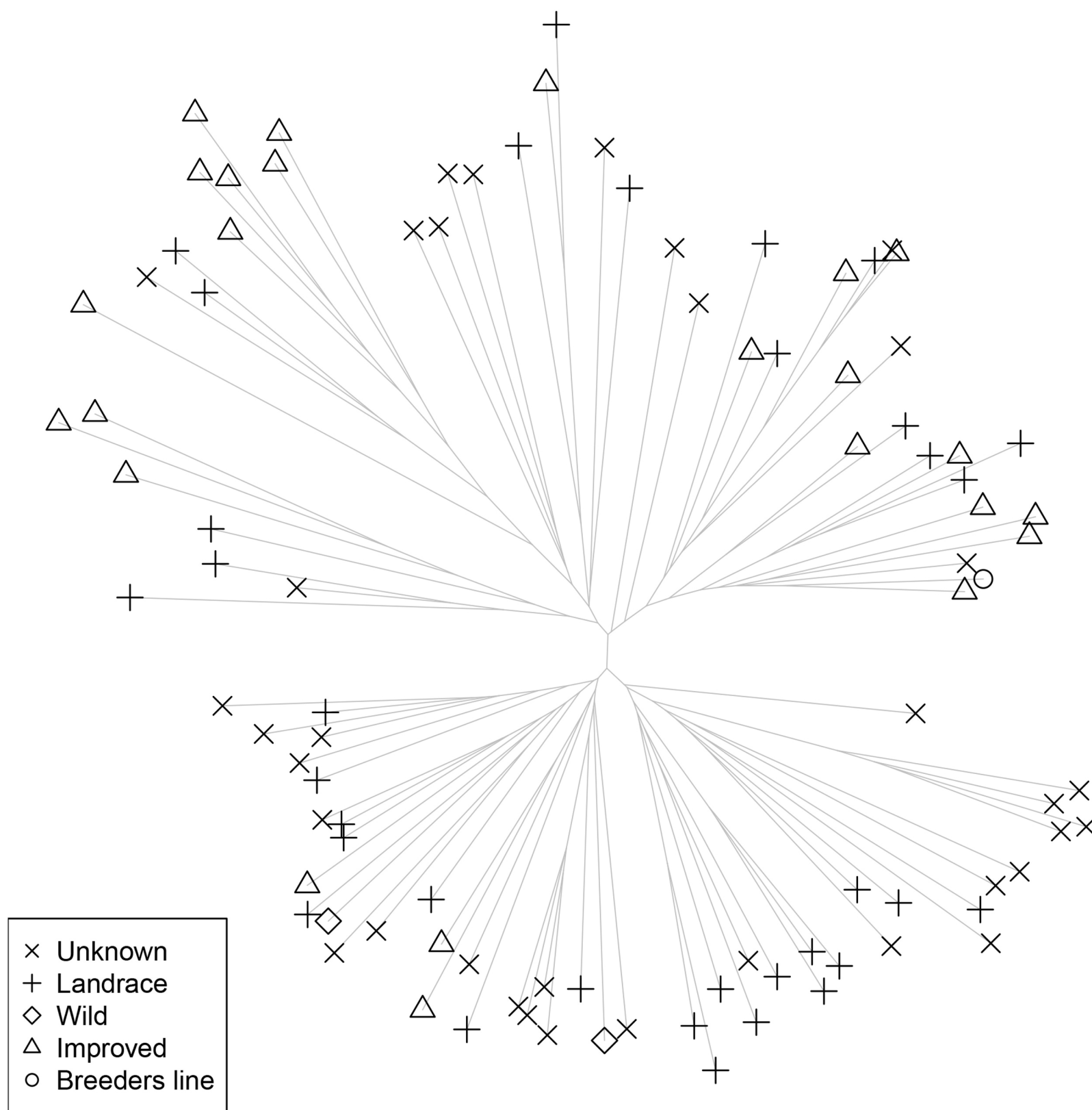**C**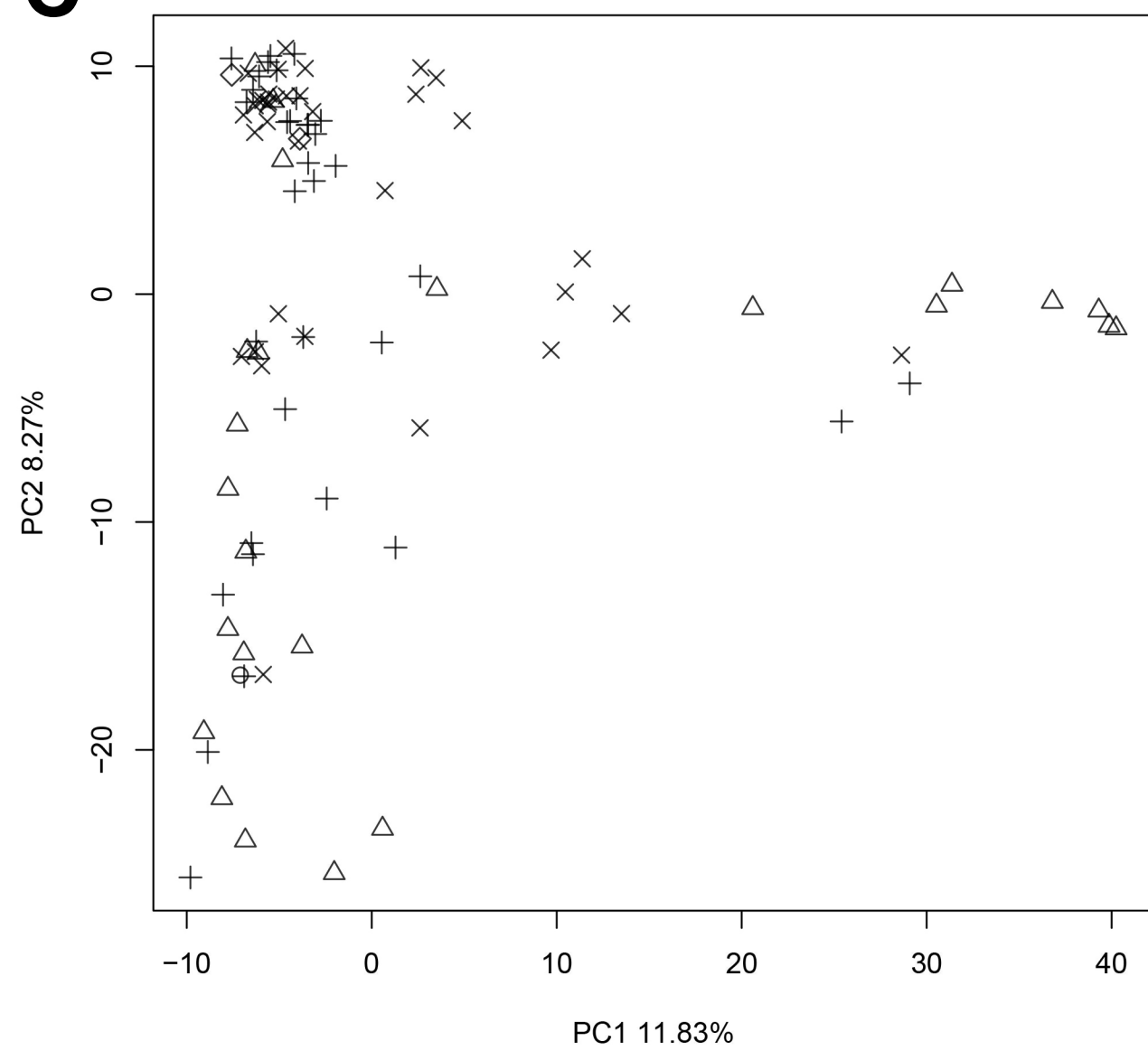

Supplement: Supplementary file 2 — Additional file 2: Supplementary Figure S1. Representative photos for proso millet seeds color classes characterized in this study. Supplementary Figure S2. Boxplots of seed trait distribution across geographical regions. Differences were analyzed using ANOVA. Supplementary Figure S3. Phenotypic and molecular analysis of proso millet accessions. (A) Principal component analysis of phenotypic diversity of seed traits and agronomic traits. (B) Phylogenetic tree derived from SNPs data. (C) Principal component analysis derived from SNPs data. Different symbols indicate type of genetic materials as shown in the legend. Supplementary Figure S4. Phenotypic diversity in the collection as reported by PC1, PC2, and PC3. Different colors on and symbols on the panels indicate region of origin and type of genetic materials as in Fig. 2 and Supplementary Figure S3. Supplementary Figure S5. PCA scores and vectors loadings for seed and agronomic traits. Percent of variance explained by each axis (PC1 = Dim1, PC2 = Dim2) is indicated in the axis titles. Vector color represents the total contribution of a given variable on the first two dimensions according to legend to the right. Supplementary Figure S6. Genotypic diversity in the collection as reported by PC1, PC2, and PCA3. Different colors on and symbols on the panels indicate region of origin and type of genetic materials as in Fig. 2 and Supplementary Figure S3. Supplementary Figure S7. Manhattan plots for GWAS on seed trait and agronomic traits. The plot shows individual SNPs across all chromosomes (x-axis) and -log10 P value of each SNP association (y-axis). The different colors indicate the 18 chromosomes of proso millet. The horizontal line shows the multiple testing threshold according to a stringent Bonferroni method. Note that different Manhattan plots are reported to different y-axis scales corresponding to the highest significance for each GWAS. Supplementary Figure S8. Quantile–Quantile (Q–Q) plots for FarmCPU model s [file 12870_2021_3111_MOESM2_ESM.zip › Fig.S3.pdf]

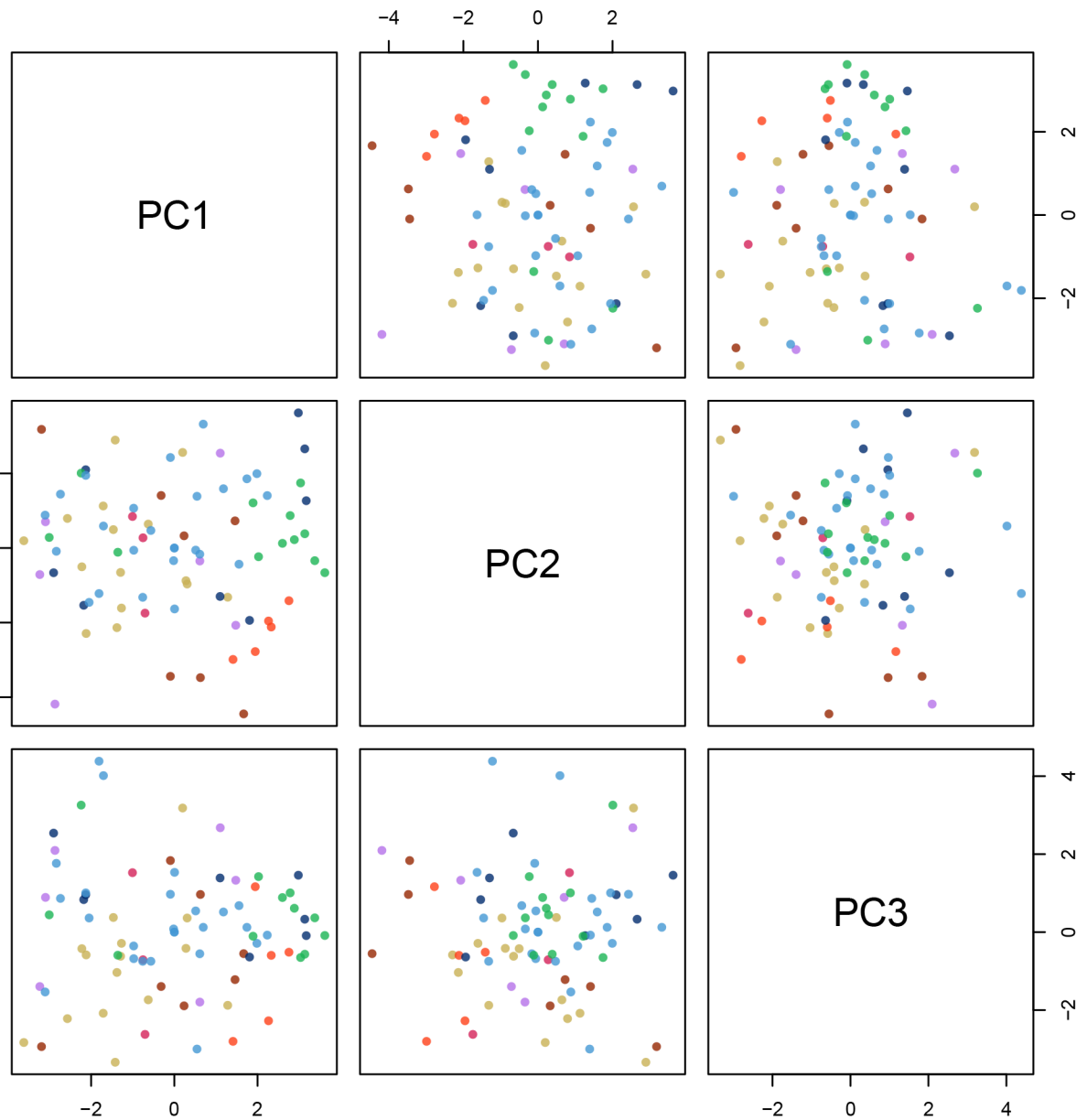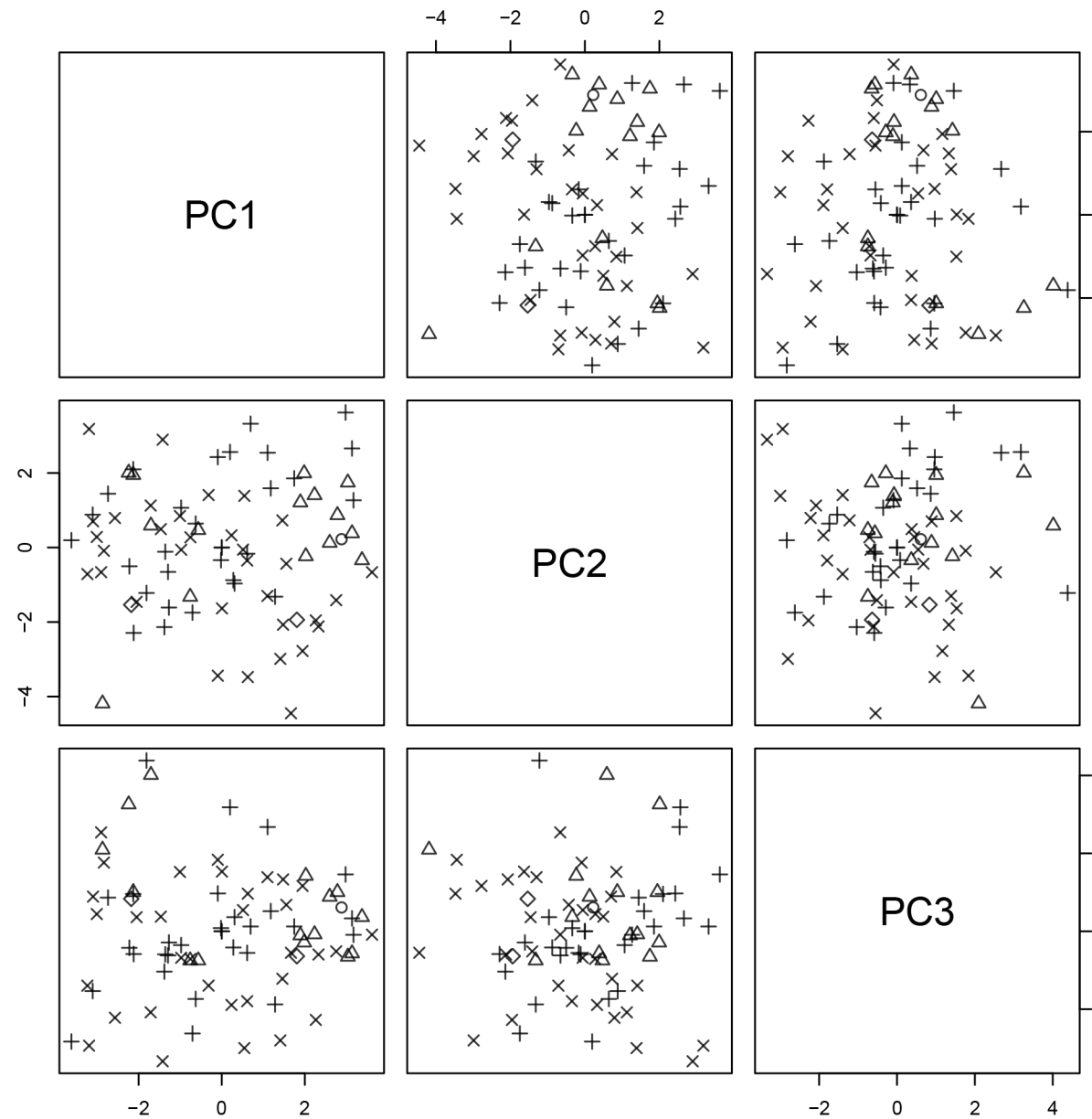

Supplement: Supplementary file 2 — Additional file 2: Supplementary Figure S1. Representative photos for proso millet seeds color classes characterized in this study. Supplementary Figure S2. Boxplots of seed trait distribution across geographical regions. Differences were analyzed using ANOVA. Supplementary Figure S3. Phenotypic and molecular analysis of proso millet accessions. (A) Principal component analysis of phenotypic diversity of seed traits and agronomic traits. (B) Phylogenetic tree derived from SNPs data. (C) Principal component analysis derived from SNPs data. Different symbols indicate type of genetic materials as shown in the legend. Supplementary Figure S4. Phenotypic diversity in the collection as reported by PC1, PC2, and PC3. Different colors on and symbols on the panels indicate region of origin and type of genetic materials as in Fig. 2 and Supplementary Figure S3. Supplementary Figure S5. PCA scores and vectors loadings for seed and agronomic traits. Percent of variance explained by each axis (PC1 = Dim1, PC2 = Dim2) is indicated in the axis titles. Vector color represents the total contribution of a given variable on the first two dimensions according to legend to the right. Supplementary Figure S6. Genotypic diversity in the collection as reported by PC1, PC2, and PCA3. Different colors on and symbols on the panels indicate region of origin and type of genetic materials as in Fig. 2 and Supplementary Figure S3. Supplementary Figure S7. Manhattan plots for GWAS on seed trait and agronomic traits. The plot shows individual SNPs across all chromosomes (x-axis) and -log10 P value of each SNP association (y-axis). The different colors indicate the 18 chromosomes of proso millet. The horizontal line shows the multiple testing threshold according to a stringent Bonferroni method. Note that different Manhattan plots are reported to different y-axis scales corresponding to the highest significance for each GWAS. Supplementary Figure S8. Quantile–Quantile (Q–Q) plots for FarmCPU model s [file 12870_2021_3111_MOESM2_ESM.zip › Fig.S4.pdf]

Variables – PCA

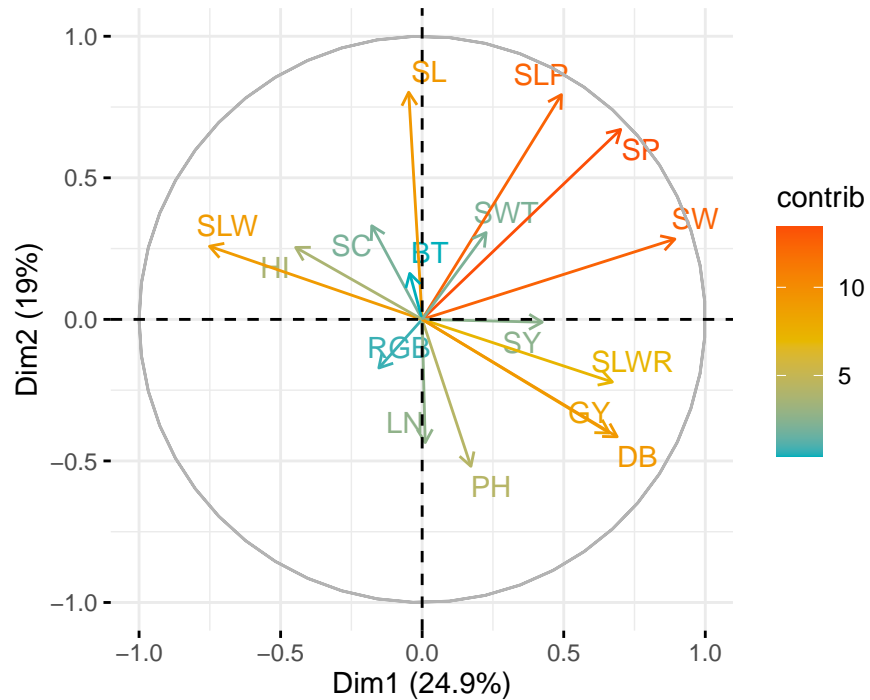

Supplement: Supplementary file 2 — Additional file 2: Supplementary Figure S1. Representative photos for proso millet seeds color classes characterized in this study. Supplementary Figure S2. Boxplots of seed trait distribution across geographical regions. Differences were analyzed using ANOVA. Supplementary Figure S3. Phenotypic and molecular analysis of proso millet accessions. (A) Principal component analysis of phenotypic diversity of seed traits and agronomic traits. (B) Phylogenetic tree derived from SNPs data. (C) Principal component analysis derived from SNPs data. Different symbols indicate type of genetic materials as shown in the legend. Supplementary Figure S4. Phenotypic diversity in the collection as reported by PC1, PC2, and PC3. Different colors on and symbols on the panels indicate region of origin and type of genetic materials as in Fig. 2 and Supplementary Figure S3. Supplementary Figure S5. PCA scores and vectors loadings for seed and agronomic traits. Percent of variance explained by each axis (PC1 = Dim1, PC2 = Dim2) is indicated in the axis titles. Vector color represents the total contribution of a given variable on the first two dimensions according to legend to the right. Supplementary Figure S6. Genotypic diversity in the collection as reported by PC1, PC2, and PCA3. Different colors on and symbols on the panels indicate region of origin and type of genetic materials as in Fig. 2 and Supplementary Figure S3. Supplementary Figure S7. Manhattan plots for GWAS on seed trait and agronomic traits. The plot shows individual SNPs across all chromosomes (x-axis) and -log10 P value of each SNP association (y-axis). The different colors indicate the 18 chromosomes of proso millet. The horizontal line shows the multiple testing threshold according to a stringent Bonferroni method. Note that different Manhattan plots are reported to different y-axis scales corresponding to the highest significance for each GWAS. Supplementary Figure S8. Quantile–Quantile (Q–Q) plots for FarmCPU model s [file 12870_2021_3111_MOESM2_ESM.zip › Fig.S5.pdf]

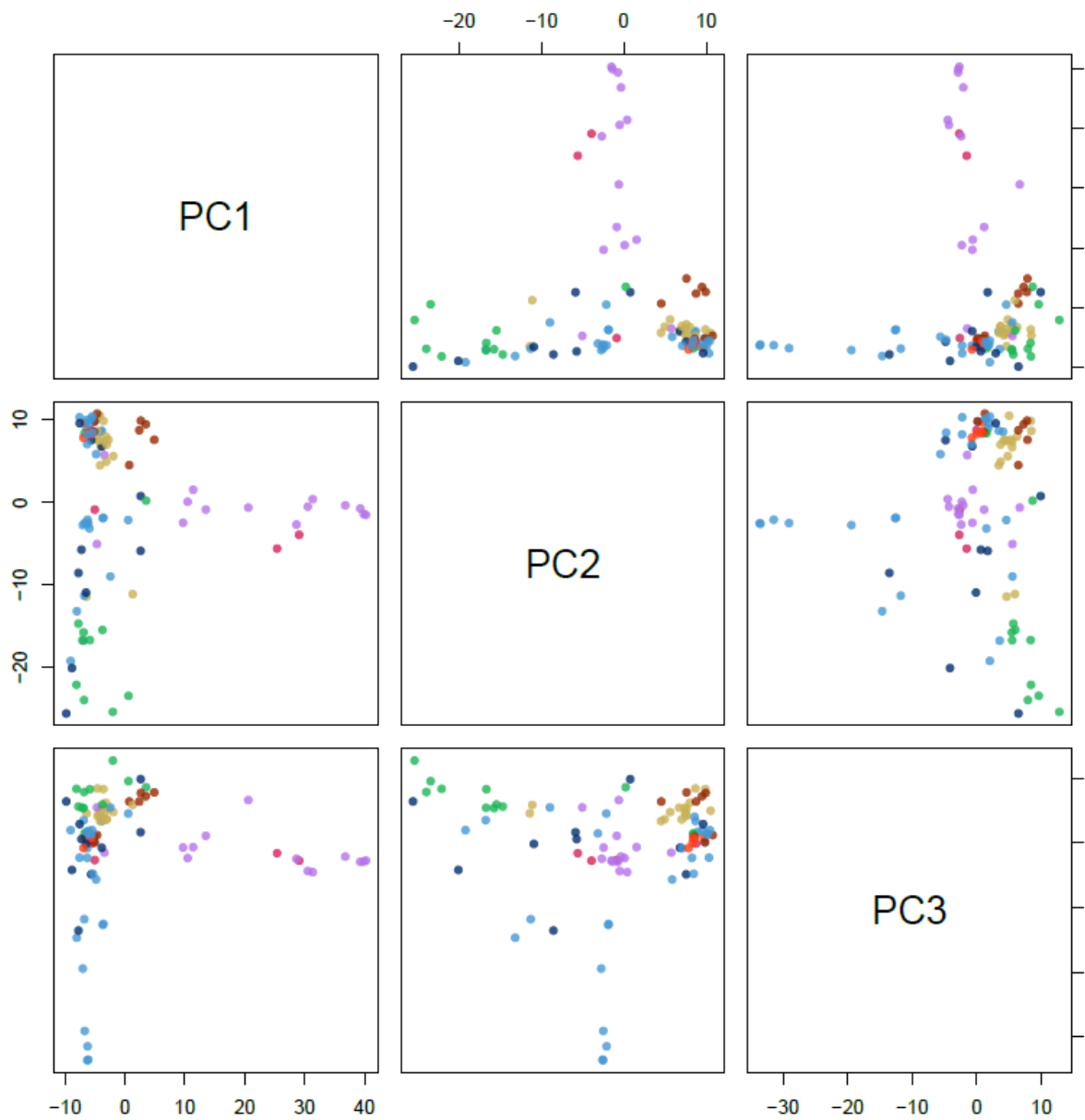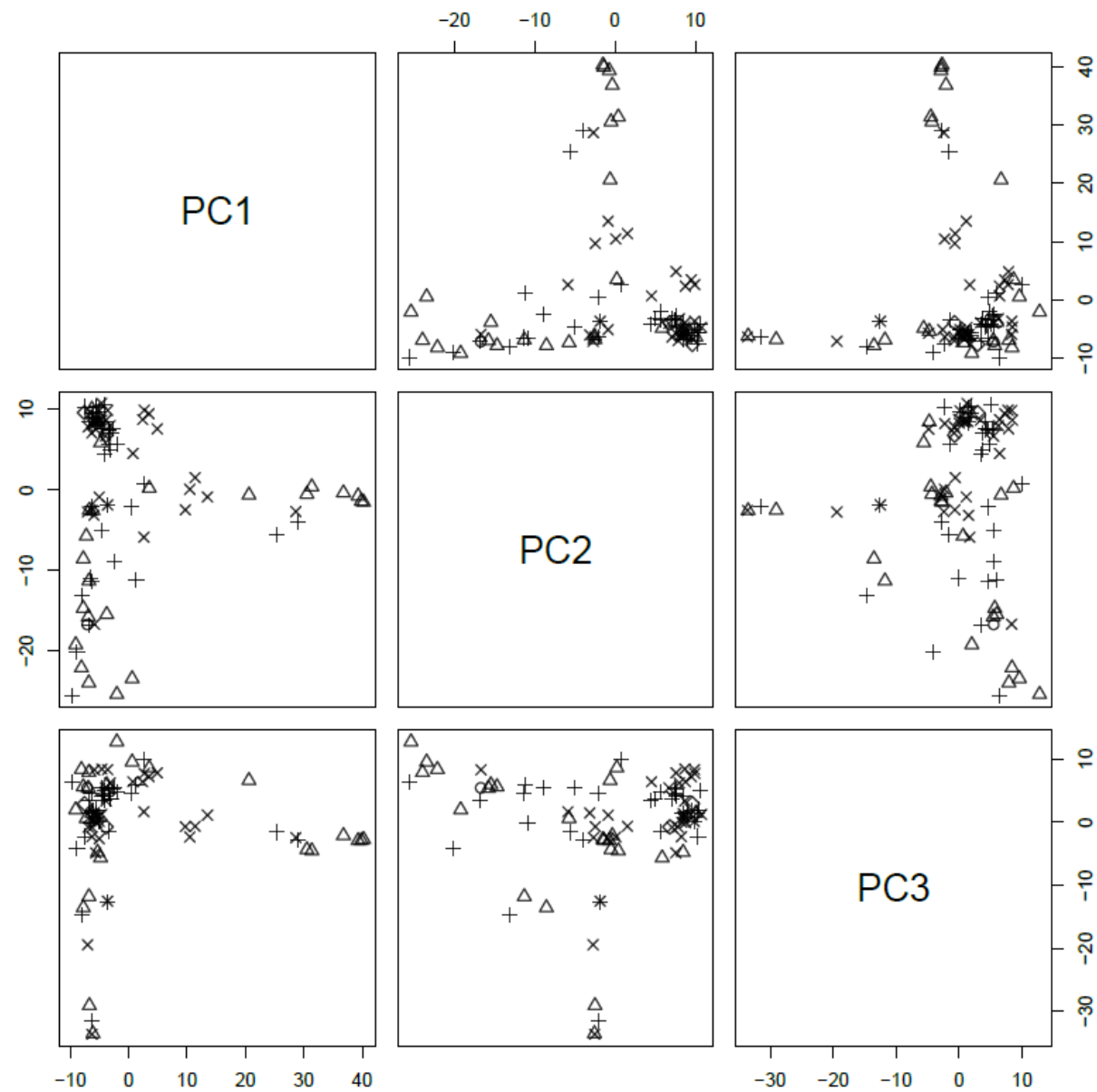

Supplement: Supplementary file 2 — Additional file 2: Supplementary Figure S1. Representative photos for proso millet seeds color classes characterized in this study. Supplementary Figure S2. Boxplots of seed trait distribution across geographical regions. Differences were analyzed using ANOVA. Supplementary Figure S3. Phenotypic and molecular analysis of proso millet accessions. (A) Principal component analysis of phenotypic diversity of seed traits and agronomic traits. (B) Phylogenetic tree derived from SNPs data. (C) Principal component analysis derived from SNPs data. Different symbols indicate type of genetic materials as shown in the legend. Supplementary Figure S4. Phenotypic diversity in the collection as reported by PC1, PC2, and PC3. Different colors on and symbols on the panels indicate region of origin and type of genetic materials as in Fig. 2 and Supplementary Figure S3. Supplementary Figure S5. PCA scores and vectors loadings for seed and agronomic traits. Percent of variance explained by each axis (PC1 = Dim1, PC2 = Dim2) is indicated in the axis titles. Vector color represents the total contribution of a given variable on the first two dimensions according to legend to the right. Supplementary Figure S6. Genotypic diversity in the collection as reported by PC1, PC2, and PCA3. Different colors on and symbols on the panels indicate region of origin and type of genetic materials as in Fig. 2 and Supplementary Figure S3. Supplementary Figure S7. Manhattan plots for GWAS on seed trait and agronomic traits. The plot shows individual SNPs across all chromosomes (x-axis) and -log10 P value of each SNP association (y-axis). The different colors indicate the 18 chromosomes of proso millet. The horizontal line shows the multiple testing threshold according to a stringent Bonferroni method. Note that different Manhattan plots are reported to different y-axis scales corresponding to the highest significance for each GWAS. Supplementary Figure S8. Quantile–Quantile (Q–Q) plots for FarmCPU model s [file 12870_2021_3111_MOESM2_ESM.zip › Fig.S6.pdf]

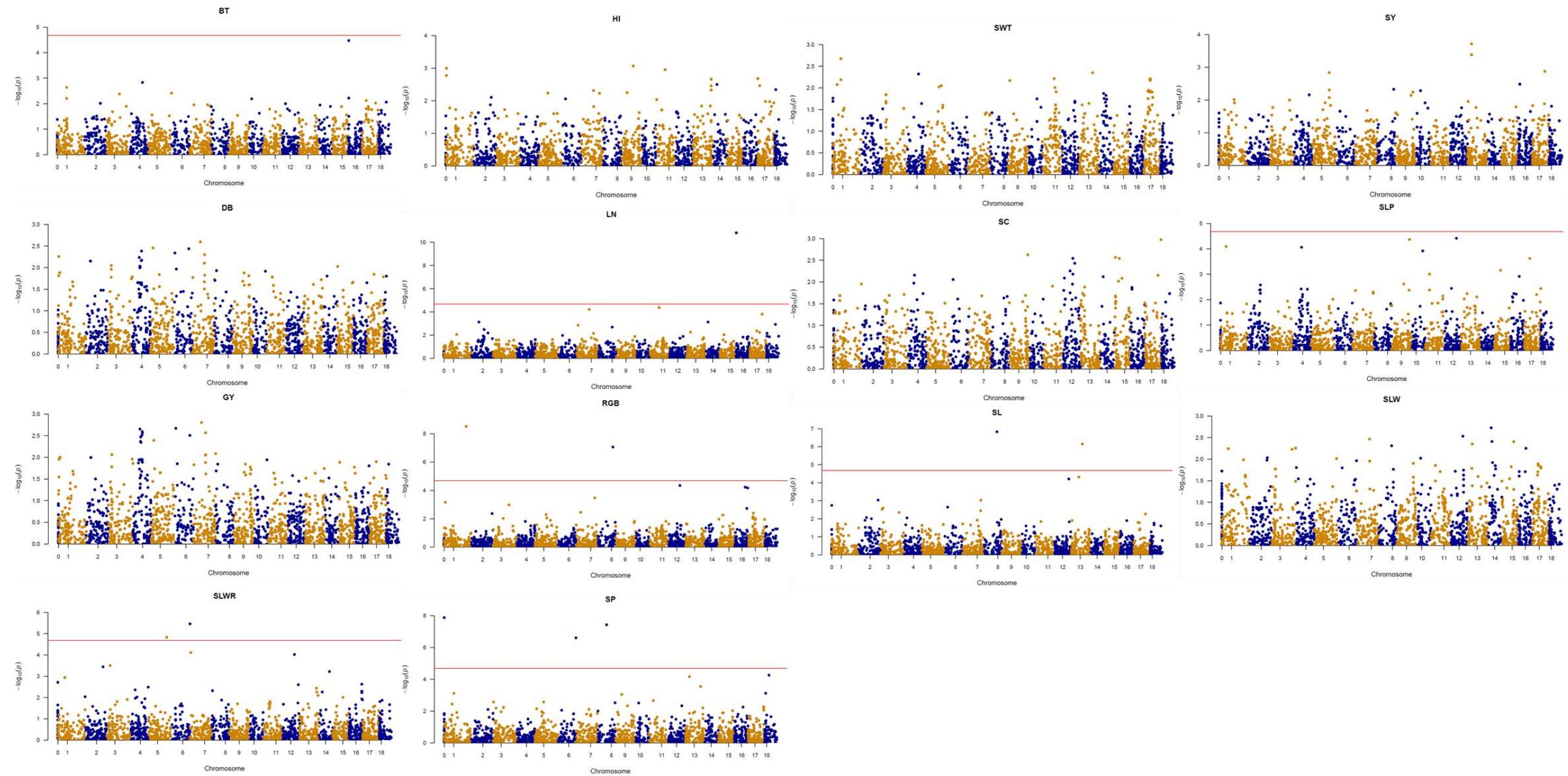

Supplement: Supplementary file 2 — Additional file 2: Supplementary Figure S1. Representative photos for proso millet seeds color classes characterized in this study. Supplementary Figure S2. Boxplots of seed trait distribution across geographical regions. Differences were analyzed using ANOVA. Supplementary Figure S3. Phenotypic and molecular analysis of proso millet accessions. (A) Principal component analysis of phenotypic diversity of seed traits and agronomic traits. (B) Phylogenetic tree derived from SNPs data. (C) Principal component analysis derived from SNPs data. Different symbols indicate type of genetic materials as shown in the legend. Supplementary Figure S4. Phenotypic diversity in the collection as reported by PC1, PC2, and PC3. Different colors on and symbols on the panels indicate region of origin and type of genetic materials as in Fig. 2 and Supplementary Figure S3. Supplementary Figure S5. PCA scores and vectors loadings for seed and agronomic traits. Percent of variance explained by each axis (PC1 = Dim1, PC2 = Dim2) is indicated in the axis titles. Vector color represents the total contribution of a given variable on the first two dimensions according to legend to the right. Supplementary Figure S6. Genotypic diversity in the collection as reported by PC1, PC2, and PCA3. Different colors on and symbols on the panels indicate region of origin and type of genetic materials as in Fig. 2 and Supplementary Figure S3. Supplementary Figure S7. Manhattan plots for GWAS on seed trait and agronomic traits. The plot shows individual SNPs across all chromosomes (x-axis) and -log10 P value of each SNP association (y-axis). The different colors indicate the 18 chromosomes of proso millet. The horizontal line shows the multiple testing threshold according to a stringent Bonferroni method. Note that different Manhattan plots are reported to different y-axis scales corresponding to the highest significance for each GWAS. Supplementary Figure S8. Quantile–Quantile (Q–Q) plots for FarmCPU model s [file 12870_2021_3111_MOESM2_ESM.zip › Fig.S7.pdf]

BT

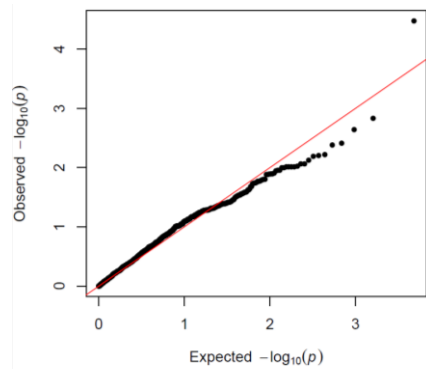

HI

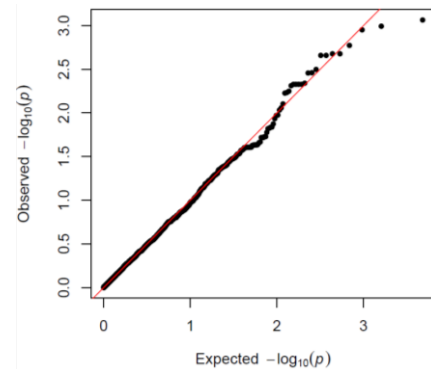

SC

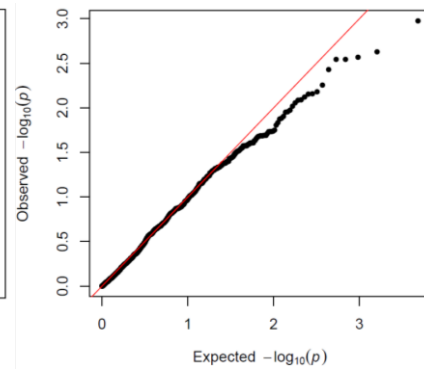

SLW

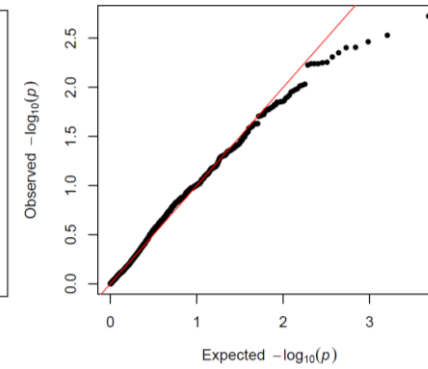

SY

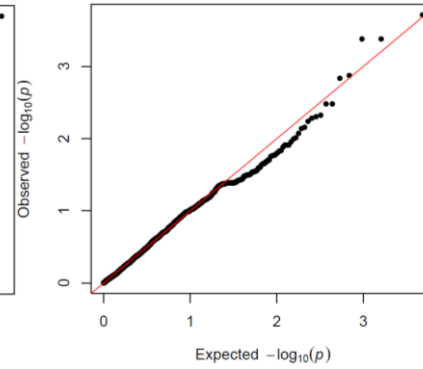

DB

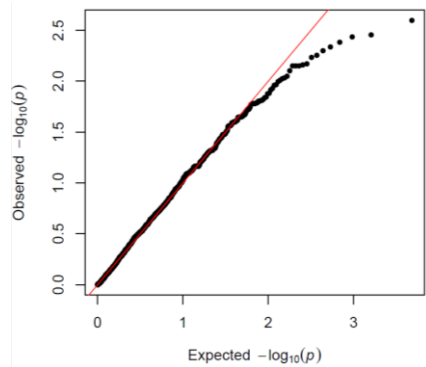

LN

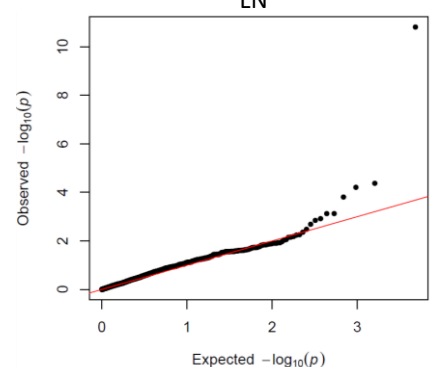

SL

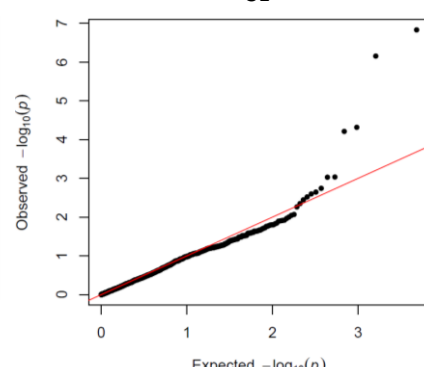

SLWR

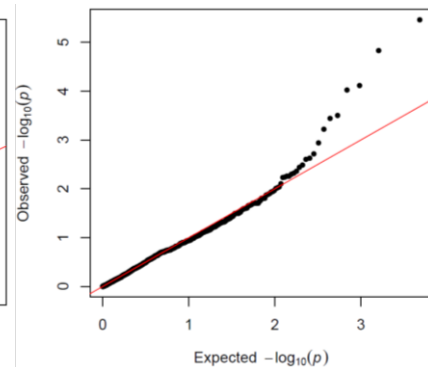

SWT

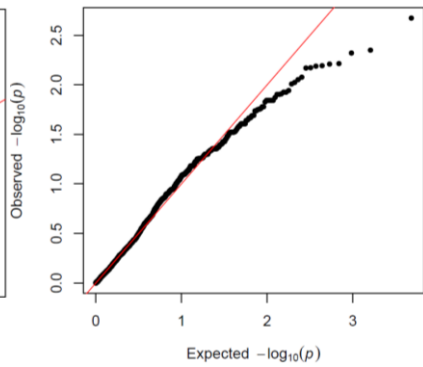

GY

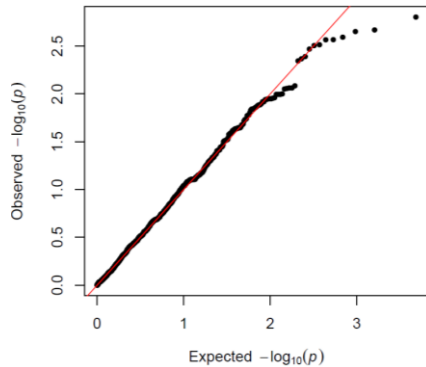

RGB

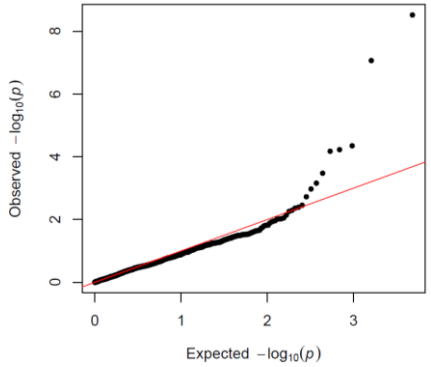

SLP

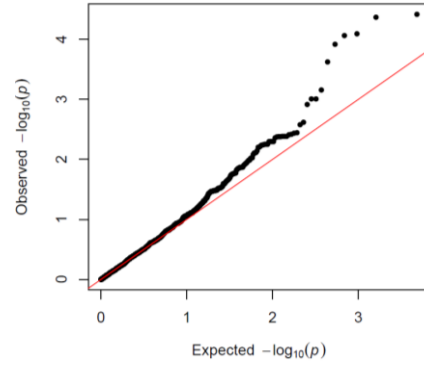

SP

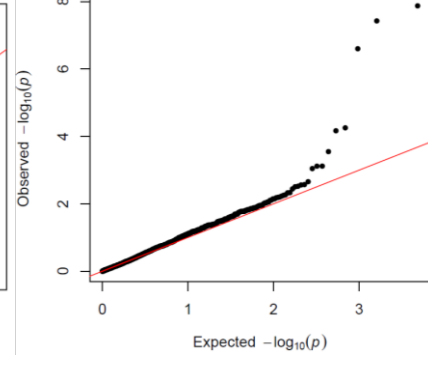

Supplement: Supplementary file 2 — Additional file 2: Supplementary Figure S1. Representative photos for proso millet seeds color classes characterized in this study. Supplementary Figure S2. Boxplots of seed trait distribution across geographical regions. Differences were analyzed using ANOVA. Supplementary Figure S3. Phenotypic and molecular analysis of proso millet accessions. (A) Principal component analysis of phenotypic diversity of seed traits and agronomic traits. (B) Phylogenetic tree derived from SNPs data. (C) Principal component analysis derived from SNPs data. Different symbols indicate type of genetic materials as shown in the legend. Supplementary Figure S4. Phenotypic diversity in the collection as reported by PC1, PC2, and PC3. Different colors on and symbols on the panels indicate region of origin and type of genetic materials as in Fig. 2 and Supplementary Figure S3. Supplementary Figure S5. PCA scores and vectors loadings for seed and agronomic traits. Percent of variance explained by each axis (PC1 = Dim1, PC2 = Dim2) is indicated in the axis titles. Vector color represents the total contribution of a given variable on the first two dimensions according to legend to the right. Supplementary Figure S6. Genotypic diversity in the collection as reported by PC1, PC2, and PCA3. Different colors on and symbols on the panels indicate region of origin and type of genetic materials as in Fig. 2 and Supplementary Figure S3. Supplementary Figure S7. Manhattan plots for GWAS on seed trait and agronomic traits. The plot shows individual SNPs across all chromosomes (x-axis) and -log10 P value of each SNP association (y-axis). The different colors indicate the 18 chromosomes of proso millet. The horizontal line shows the multiple testing threshold according to a stringent Bonferroni method. Note that different Manhattan plots are reported to different y-axis scales corresponding to the highest significance for each GWAS. Supplementary Figure S8. Quantile–Quantile (Q–Q) plots for FarmCPU model s [file 12870_2021_3111_MOESM2_ESM.zip › Fig.S8.pdf]
